# Puberty Status Modifies the Effects of Genetic Variants, Lifestyle Factors and Their Interactions on Adiponectin: The BCAMS Study

**DOI:** 10.3389/fendo.2021.737459

**Published:** 2021-12-24

**Authors:** Yunpeng Wu, Ling Zhong, Ge Li, Lanwen Han, Junling Fu, Yu Li, Lujiao Li, Qian Zhang, Yiran Guo, Xinhua Xiao, Lu Qi, Ming Li, Shan Gao, Steven. M. Willi

**Affiliations:** ^1^ Department of Endocrinology, National Health Commission (NHC) Key Laboratory of Endocrinology, Peking Union Medical College Hospital, Chinese Academy of Medical Sciences and Peking Union Medical College, Beijing, China; ^2^ Department of Endocrinology, Chaoyang Hospital, Capital Medical University, Beijing, China; ^3^ Center for Applied Genomics, Children’s Hospital of Philadelphia, Philadelphia, PA, United States; ^4^ Center for Data Driven Discovery in Biomedicine, Children’s Hospital of Philadelphia, Philadelphia, PA, United States; ^5^ Department of Epidemiology, School of Public Health and Tropical Medicine, Tulane University, New Orleans, LA, United States; ^6^ Division of Endocrinology, The Children’s Hospital of Philadelphia, Perelman School of Medicine, University of Pennsylvania, Philadelphia, PA, United States

**Keywords:** adiponectin, puberty, diet items, genetic variants, gene-by-lifestyle interaction

## Abstract

**Background:**

Hypoadiponectinemia has been associated with various cardiometabolic disease states. Previous studies in adults have shown that adiponectin levels were regulated by specific genetic and behavioral or lifestyle factors. However, little is known about the influence of these factors on adiponectin levels in children, particularly as mitigated by pubertal development.

**Methods:**

We performed a cross-sectional analysis of data from 3,402 children aged 6-18 years from the Beijing Child and Adolescent Metabolic Syndrome (BCAMS) study. Pubertal progress was classified as prepubertal, midpuberty, and postpuberty. Six relevant single nucleotide polymorphisms (SNPs) were selected from previous genome-wide association studies of adiponectin in East Asians. Individual SNPs and two weighted genetic predisposition scores, as well as their interactions with 14 lifestyle factors, were analyzed to investigate their influence on adiponectin levels across puberty. The effect of these factors on adiponectin was analyzed using general linear models adjusted for age, sex, and BMI.

**Results:**

After adjustment for age, sex, and BMI, the associations between adiponectin levels and diet items, and diet score were significant at prepuberty or postpuberty, while the effect of exercise on adiponectin levels was more prominent at mid- and postpuberty. Walking to school was found to be associated with increased adiponectin levels throughout puberty. Meanwhile, the effect of *WDR11-FGFR2*-rs3943077 was stronger at midpuberty (*P* = 0.002), and *ADIPOQ*-rs6773957 was more effective at postpuberty (*P* = 0.005), while *CDH13*-rs4783244 showed the strongest association with adiponectin levels at all pubertal stages (all *P* < 3.24 × 10^-15^). We further found that effects of diet score (*P*_interaction_ = 0.022) and exercise (*P*_interaction_ = 0.049) were stronger in children with higher genetic risk of hypoadiponectinemia, while higher diet score and exercise frequency attenuated the differences in adiponectin levels among children with different genetic risks.

**Conclusions:**

Our study confirmed puberty modulates the associations between adiponectin, and genetic variants, lifestyle factors, and gene-by-lifestyle interactions. These findings provide new insight into puberty-specific lifestyle suggestions, especially in genetically susceptible individuals.

## Introduction

Childhood obesity has emerged as a global public health problem, in part due to its association with cardiometabolic disease (1). In China, overweight and obesity have increased rapidly over the past four decades, and the prevalence of overweight and obesity among children aged 6-17 and under 6 is 19% and 10.4% respectively, which has become a major challenge for the country’s healthcare system (2, 3). Yet, the mechanisms responsible for obesity’s contribution to cardiometabolic risk, like adipocyte metabolic dysregulation, remain unclear (4). Adipose tissue, as an endocrine organ, secretes many peptide hormones, termed “adipokines”, that affect systemic metabolism (5). Among the most abundant adipokines, adiponectin is specifically expressed in differentiated adipocytes and exhibits anti-atherogenic, anti-inflammatory and anti-diabetic properties (6). Low adiponectin levels, known as hypoadiponectinemia, and a marker of adipose tissue dysfunction, and the condition that is common in obesity (7), have been robustly associated with an increased risk of insulin resistance, diabetes, cardiovascular diseases, and certain kinds of cancer (8, 9). As a result, adiponectin is regarded as a protective molecule and a potentially novel therapeutic target for diabetes and related diseases (10). Some diabetes drugs, such as rosiglitazone, operate partially by increasing circulating adiponectin levels (11).

Recently, several genetic (12, 13) and environmental factors (14–17) that influence adiponectin levels have been reported in adult populations; however, the understanding of these relationships in children, especially during puberty, is still quite limited (18). Since many adult diseases have their origins in childhood (19), it is important to identify the factors that influence adiponectin levels during pediatric development. As puberty is a period through which the body changes physically, being a physiological process leading to the maturation of children (20) and sex differences of adiponectin seem to develop during the development of puberty (21), we propose that the influences which genetic and environmental factors (as well as gene-environment interactions) exert upon adiponectin levels are mitigated by pubertal stage and that this modulating effect of puberty is mediated through adipose tissue development. Therefore, leveraging the large cohort within the Beijing Child and Adolescent Metabolic Syndrome (BCAMS) study (22), we aimed to examine the effect of pubertal stage upon adiponectin’s association with specific gene variants, lifestyle influences, and gene-environment interactions.

## Materials and Methods

### Subjects

The BCAMS study, which has been described in detail elsewhere (22, 23), is an ongoing cohort study of obesity and related metabolic abnormalities in a representative sample of school-age children (n = 19,593, aged 6-18 y, 50% boys) recruited from the Beijing area between April and October 2004. Within this cohort, 4,500 subjects were identified as being at risk of metabolic syndrome (MS), based on at least one of the following criteria: 1) overweight, as defined by body mass index (BMI) percentile; 2) increased total cholesterol (TC) ≥ 5.2 mmol/L; 3) triglycerides (TG) ≥ 1.7 mmol/L; and 4) fasting blood glucose (FBG) ≥ 5.6 mmol/L based on finger capillary blood tests. Next, all children at risk of MS, together with a parallel normal sample of 1,024 schoolchildren, were invited to participate in a further medical examination including venipuncture-based blood tests. In total, 3,506 subjects, including 2,525 subjects with MS risk, ultimately completed the further clinical examination. Thus, the presence of pediatric MS based on clinical examination was defined by the presence of three or more of the following five components (22, 23) (1): central obesity defined as ≥90th percentile for age and gender (2); elevated systolic and/or diastolic blood pressure ≥90th percentile for age, sex and height (3); hypertriglyceridemia defined as TG ≥1.24 mmol/L (4); low high-density lipoprotein (HDL) cholesterol defined as <1.03 mmol/L; and (5) hyperglycemia defined as FBG ≥ 5.6 mmol/L. Accordingly, in the current study, we used the cross-sectional data of 3,402 participants (including 2,112 subjects with MS risk and 1,290 subjects without MS risk based on clinical evaluation), who completed the examination of adiponectin levels, genotype, and lifestyle factors in 2004 (**Supplementary Figure S1** and **Supplementary Table S1**).

### Anthropometric Measurements and Pubertal Stages

The subjects’ height and weight were measured according to our standard protocol (22, 24). Height in centimeters was measured without shoes to the nearest 0.1 cm. Bodyweight was measured to the nearest 0.1 kg (light indoor clothing, without shoes) using a calibrated electronic scale. BMI was calculated as weight (kg) divided by height squared (m^2^). Age- and sex-specific BMI percentiles were used to define overweight (85^th^) and obesity (95^th^) following the Working Group for Obesity in China (25). Puberty stages were assessed by two pediatricians of the same gender, based on Tanner’s stages of breast development in girls and testicular volume in boys, in line with Marshall and Tanner (26). The categories of puberty were defined as pre-puberty (Tanner stage I), mid-puberty (Tanner stage II-III), and post-puberty (Tanner stage ≥ IV) (26).

### Lifestyle Description

Fourteen lifestyle factors (27), including walking to school, frequency of exercise, duration of habitual sleep, and eleven dietary habits were selected for examination in this study (**Table 1**). And the comparison of lifestyle factors between subjects with risk of MS and without risk of MS was listed in **Supplementary Table S1**. Surveys were conducted retrospectively and participants were asked how often on average they had consumed each food in the previous month. Each dietary item was scored on a 5-point scale according to the frequency categories from “seldom or never” to “> 5 times per week “, with ascending values for favorable foods (1 for “seldom or never” and 5 for “> 5 times per week”) and vice versa, according to the direction of their linear associations with adiponectin levels. The diet score was generated by summing all the selected item scores, with a higher diet score indexing predisposition to higher adiponectin levels. Effective exercise was deemed as exercise lasting longer than 30 minutes for extracurricular physical activities such as cycling, running, swimming, dancing, and team sports. Transportation refers to “walking to school” which was recorded and reclassified into two modes, ‘walking’ and ‘non-walking’.

**Table 1:** Comparison of lifestyle factors among the various pubertal stages.

	Entire Population^c^	Prepuberty^cd^	Midpuberty^cd^	Postpuberty^cd^	P-value
N	3402	1092	1076	1234	
Age (years)	13 ± 3	9 ± 2	13 ± 2	15 ± 2	**<0.001**
**Sex**					
Male	1707	755	649	303	**<0.001**
Female	1695	337	427	931	**<0.001**
BMI (kg/m^2^)	21.8 ± 4.9	19.9 ± 4.6	21.9 ± 4.9	23.4 ± 4.7	**<0.001**
Normal weight, %	47.2	45.1	48.7	47.7	**<0.001**
Ln-adiponectin (μg/ml) ^a^	1.7 ± 0.6	1.9 ± 0.6	1.6 ± 0.6	1.6 ± 0.6	**<0.001**
Diet^b^					
Breakfast	4.4 ± 1.3	4.7 ± 1	4.4 ± 1.3	4.1 ± 1.3	**<0.001**
Bean	2.7 ± 1.4	2.8 ± 1.4	2.8 ± 1.4	2.5 ± 1.4	**<0.001**
Meat	3.7 ± 1.5	3.7 ± 1.5	3.8 ± 1.4	3.5 ± 1.5	**<0.001**
Sea food	2.2 ± 1.3	2.5 ± 1.3	2.2 ± 1.3	2.0 ± 1.2	**<0.001**
Diary	3.8 ± 1.6	4.2 ± 1.4	4.7 ± 1.6	3.5 ± 1.7	**<0.001**
Vegetable	4.8 ± 0.8	4.7 ± 0.8	4.8 ± 0.8	4.8 ± 0.6	**<0.001**
Fruit	4.1 ± 1.3	4.1 ± 1.3	4.0 ± 1.4	4.1 ± 1.3	0.341
Fast food	1.4 ± 0.8	1.5 ± 0.9	1.4 ± 0.8	1.4 ± 0.7	**<0.001**
Soft drink	2.5 ± 1.5	2.3 ± 1.4	2.6 ± 1.5	2.6 ± 1.5	**<0.001**
Fried food	2.0 ± 1.2	1.9 ± 1.1	2.0 ± 1.2	2.0 ± 1.3	**0.017**
Snacks	2.6 ± 1.5	2.4 ± 1.5	2.6 ± 1.6	2.6 ± 1.5	**0.008**
Exercise^b^	3.4 ± 1.3	3.6 ± 1.2	3.5 ± 1.3	3.1 ± 1.4	**<0.001**
Walking to school, %	57	66	62	45	**<0.001**
Sleep duration (h/day)	8.5 ± 1.2	9.1 ± 0.9	8.6 ± 1.1	8.0 ± 1.2	**<0.001**

a Adiponectin levels were natural logarithmically (ln) transformed.

b The values of the diet items and exercise were encoded as “seldom or never” = 1; “1 time/2 weeks” = 2; “1-2 times per week” = 3; “3-5 times per week” = 4; “> 5 times per week” = 5.

c Data are expressed as the mean ± SD or n (%).

d Prepuberty: Tanner stage I; Midpuberty: Tanner stage II-III; Postpuberty: Tanner stage ≥ IV.

Boldface type indicates nominally significant values (P < 0.05).

### Laboratory Measurements

Venous blood samples were collected after 12 h of fasting. The adiponectin concentration was measured using a monoclonal antibody-based enzyme-linked immunosorbent assay (28). The intra- and interassay coefficients of variation were < 5.4% and 8.5%, respectively.

### Single Nucleotide Polymorphism Selection and Genotyping

Genomic DNA was isolated from peripheral white blood cells using QIAamp DNA blood midikits (Qiagen). Genotyping was carried out on a Sequenom Mass Array iPLEX genotyping platform by BioMiao Biological Technology Co., Ltd (29). All these SNPs had genotyping efficiency >0.95 and were in Hardy-Weinberg equilibrium with p-value >0.008 (0.05/6). We selected six SNPs showing strong associations with adiponectin levels in previous genome-wide association studies (GWASs) (12, 13). Among them, five SNPs (*CDH13*-rs4783244, *ADIPOQ*-rs10937273, *PEPD*-rs889140, *CMIP*-rs2925979, and *WDR11-FGFR2*-rs3943077) were identified in an East Asian adult population (12) to have the five strongest associations with adiponectin levels (*P* < 10^-10^ for each of the five SNPs); while another SNP, *ADIPOQ*-rs6773957, was identified in a European population (13) and was included because it is located at the 3’ UTR of *ADIPOQ*, a very important regulatory area of the gene. Details of the SNPs are listed in **Supplementary Table S2**.

### Construction of Genetic Predisposition Scores

As the effects of the SNPs were dramatically different, we used weighted rather than unweighted genetic predisposition scores (wGPSs) to evaluate the genetic structure of the children. wGPSs were calculated by summing the scores of the six SNPs, each of which was weighted using the mean of linear regression β from our study (wGPS_all_-_BCAMS_) or that of published GWASs (wGPS_all_-_GWAS_) for adiponectin levels (12, 13). *CDH13*-rs4783244 was found to be the strongest modulator of adiponectin levels in the current study, explaining at least 10-fold higher adiponectin levels than the others (**Supplementary Table S2**). We generated two GPSs that excluded *CDH13*-rs4783244, namely, wGPS_no CDH13-GWAS_ and wGPS_no CDH13_-_BCAMS_, to evaluate the association of genetic structure with adiponectin expression. For each participant *j* (*j* = 1, 2,…, 3405), we calculated wGPSs using the following equation:


$$wGPS_{n}^{\left(j\right)}=n\times \frac{\sum_{i=1}^{n}\beta_{i}^{\left(j\right)}\cdot EA_{i}^{\left(j\right)}}{\sum_{i=1}^{n}\beta_{i}^{\left(j\right)}},$$

where *n* is 6 (i.e., wGPS_all_; including all six SNPs) or 5 (i.e., wGPS_no CDH13_; all SNPs but *CDH13*-rs4783244), *β_i_* is the *β* coefficient of each SNP for adiponectin levels (natural logarithm-transformed) adjusted for age, sex, and BMI, and *EA_i_* is the number of effect alleles (0, 1 or 2) in each SNP. Thus, wGPS_all_ ranged from 0 to 12, while wGPS_no CDH13_ ranged from 0 to 10. However, there were no significant differences between the results of wGPS_all_-_BCAMS_ and wGPS_all_-_GWAS_ or between wGPS_no CDH13_-_GWAS_ and wGPS_no CDH13_-_BCAMS_ in the current study. Considering that SNP-adiponectin associations could be different between children and adults, we only report the results for wGPS_all_-_BCAMS_ and wGPS_no CDH13_-_BCAMS_ in the current pediatric study, in the form of wGPS_all_ and wGPS_no CDH13_ for brevity.

### Statistical Analysis

All analyses were performed using SPSS version 22.0 software for Windows (SPSS Inc.) (30). Adiponectin levels were natural logarithm transformed for analysis. We assigned a score of 1-5 (“seldom or never” = 1; “1 time every 2 weeks” = 2; “1-2 times per week” = 3; “3-5 times per week” = 4; “> 5 times per week” = 5) to each lifestyle factor to facilitate the analyses. The exception was transportation (non-walking = 0, walking = 1). The results are expressed as the mean ± SD or mean (95% CI) unless otherwise stated. We used ANOVA, ANCOVA, and *t* tests to compare the values of factors between different puberty groups. We performed a linear regression adjusted for confounders including age, sex, and BMI, to evaluate the associations of adiponectin with SNPs, GPSs, and lifestyle factors. Furthermore, gene-lifestyle interactions on adiponectin were tested using linear regression models by including the interaction terms (e.g. diet*genotype) in these models. The β coefficient, which reflects the change in the serum adiponectin concentration, was used to report the effects of genetic variants and lifestyle on adiponectin levels. Associations between SNPs and adiponectin levels were assessed using an additive model in which a score of 0, 1, or 2 was assigned to genotypes according to the number of effect alleles. An SNP association was considered statistically significant if the resulting *P*-value was less than the Bonferroni-corrected significance threshold of 0.05/6 = 0.008. We only tested the interactions between gene variants and lifestyle factors that were statistically significantly associated with adiponectin levels, and then stratified analyses were conducted to observe effect modification. The gene-by-lifestyle interaction was considered to be significant if the resulting *P*-value was less than the Bonferroni-corrected significance threshold of 0.05/9 = 0.006 and considered to be “nominally significant” if the *P-*value between 0.006 and 0.05.

## Results

### Population Characteristics

**Figure 1:**
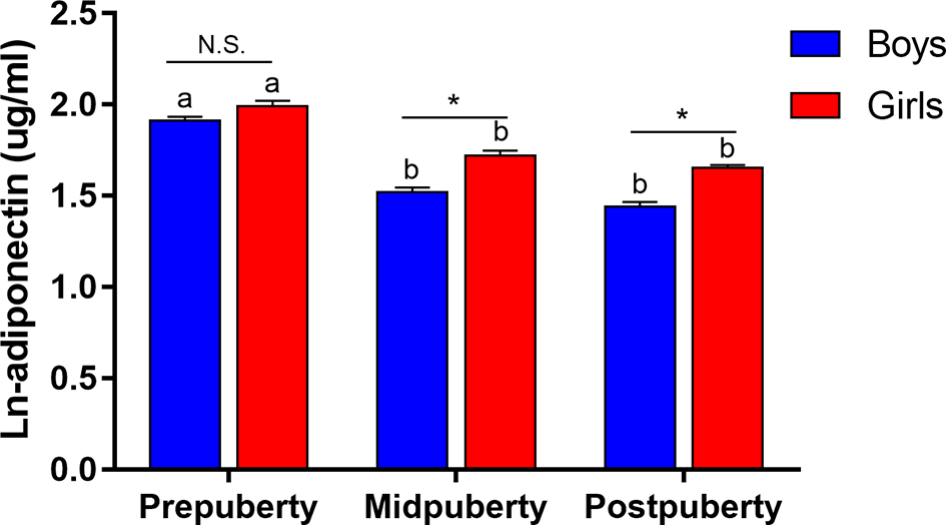
Changes in ln-adiponectin levels during puberty according to sex. Data were shown as mean and SE. Puberty status was reclassified into prepuberty (Tanner stage I), midpuberty (Tanner stage II-III) and postpuberty (Tanner stage ≥ IV). Difference between boys and girls in the same pubertal group was indicated *P < 0.001 and N.S. as non-significant value after adjusted for age. Difference between diverse pubertal groups of the same sex was indicated as different letters with P < 0.05 after adjusted for age, that was, difference between pubertal groups of the same sex with the same letter were not statistically significant.

Among the 3,402 children in our study, 32% were prepubertal, 32% were midpubertal, and 36% were postpubertal (**Table 1**). Except for fruit intake, the examined lifestyle factors differed significantly among children at different pubertal stages. Children at an advanced pubertal stage generally exhibited a higher BMI and lower adiponectin levels. **Figure 1** highlights the adiponectin levels among the different groups according to sex and puberty status. Compared with prepuberty, adiponectin levels significantly decreased after the onset of puberty, and adiponectin levels were higher in girls after adjusting for age.

### Genotypic Influences at Different Pubertal Stages

**Figure 2:**
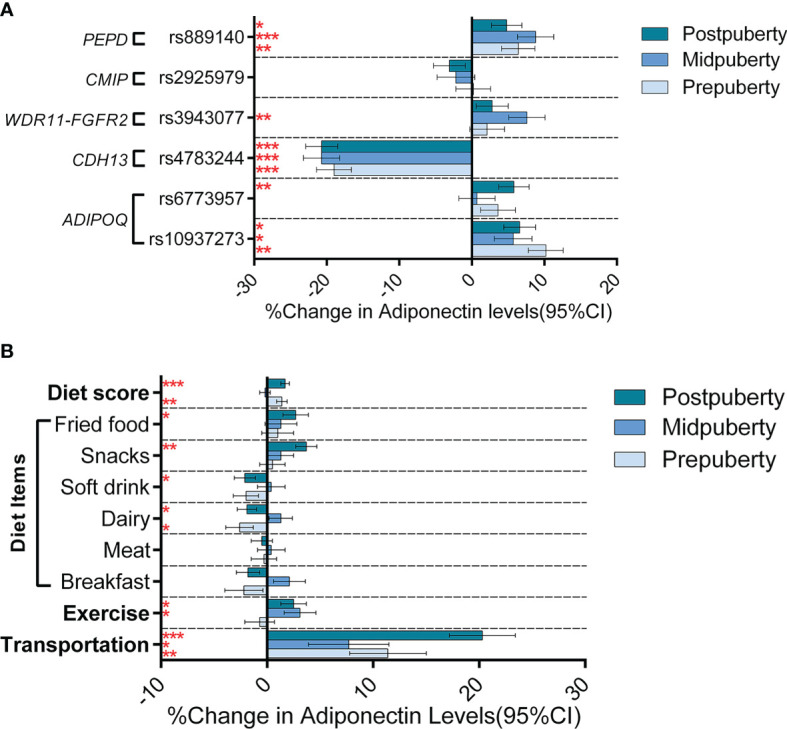
Genetic and lifestyle associations with adiponectin levels according to the different puberty stages. Figure **(A)** shows the effect (histograms) and SEs (error bar) of SNPs on adiponectin levels (% change in adiponectin levels per effect allele) at different puberty stages. Figure **(B)** shows the effect (histograms) and SEs (error bar) of lifestyle factors on adiponectin levels (% change in adiponectin levels when walking to school for the transportation variable and % change in adiponectin levels per assigned score increase for other variables) at different puberty stages. The results were adjusted for age, sex, and BMI. *P < 0.05; ** *P* < 0.05/6 = 0.008 (after Bonferroni correction); *** *P* < 0.001.

**Supplementary Table S2** shows the genetic information for the six selected SNPs. After controlling for age, sex, and BMI, five of these loci significantly influenced adiponectin levels: *CDH13*-rs4783244, *ADIPOQ*-rs10937273, *PEPD*-rs889140, *ADIPOQ*-rs6773957, and *WDR11-FGFR2*-rs3943077, explaining 5.2%, 0.7%, 0.5%, 0.2%, and 0.2% of the total variance, respectively. As for the two wGPSs, both wGPS_all_ and wGPS_no CDH13_ were strongly positively associated with adiponectin levels and explained 7.1% and 1.9% of the total variance, respectively. **Table 2** outlines the associations between the SNPs and adiponectin levels according to pubertal status in the current study. The changes in the strength of these associations during puberty after adjustment for age, sex and BMI are graphically highlighted in **Figure 2A**. The association of *ADIPOQ*-rs6773957 with adiponectin levels was only significant at postpuberty [β = 0.058 (0.017, 0.099), *P* = 0.005], while *WDR11-FGFR2*-rs3943077 presented a significant effect on adiponectin levels at midpuberty [β = 0.076 (0.027, 0.125), *P* = 0.002]. The other three SNPs, namely, *ADIPOQ*-rs10937273, *CDH13*-rs4783244, and *PEPD*-rs889140, together with wGPS_all_ and wGPS_no CDH13_, were associated with adiponectin levels throughout puberty. Most notably, among the six SNPs, *CDH13*-rs4783244 showed the strongest association with adiponectin levels at all pubertal stages.

**Table 2:** SNPs’ effect on adiponectin levels adjusted for age, sex, and BMI.

SNP/GPS	Prepuberty^b^		Midpuberty^b^		Postpuberty^b^	
	β (95%CI) ^c^	*P ^c^*	β (95%CI) ^c^	*P ^c^*	β (95%CI) ^c^	*P* ^c^
*ADIPOQ*- rs10937273	0.102(0.055 to 0.149)	**2.34×10^-05^**	0.057(0.010 to 0.104)	**0.028**	0.066(0.023 to 0.109)	**0.002**
*ADIPOQ*- rs6773957	0.036(-0.011 to 0.083)	0.131	0.007(-0.042 to 0.056)	0.781	0.058(0.017 to 0.099)	**0.005**
*CDH13*- rs4783244	-0.190(-0.237 to -0.143)	**3.24×10^-15^**	-0.207(-0.256 to -0.158)	**5.85×10^-16^**	-0.207(-0.250 to -0.164)	**2.46×10^-20^**
*WDR11-FGFR2-* rs3943077	0.021(-0.026 to 0.068)	0.377	0.076(0.027 to 0.125)	**0.002**	0.028(-0.015 to 0.071)	0.193
*CMIP*- rs2925979	0.002(-0.045 to 0.049)	0.938	-0.022(-0.073 to 0.029)	0.405	-0.031(-0.074 to 0.012)	0.152
*PEPD*- rs889140	0.064(0.019 to 0.109)	**0.006**	0.088(0.039 to 0.137)	**5.09×10^-4^**	0.048(0.007 to 0.089)	**0.025**
*wGPS-GWAS*	0.072 (0.057 to 0.087)	**4.28×10^-20^**	0.080(0.064 to 0.096)	**3.39×10^-21^**	0.077(0.063 to 0.091)	**7.14×10^-26^**
*wGPS-BCAMS*	0.071(0.056 to 0.085)	**2.53×10^-21^**	0.076(0.061 to 0.092)	**1.75×10^-21^**	0.074(0.061 to 0.087)	**3.33×10^-26^**
wGPS-GWAS(no CDH13)	0.044 (0.024 to 0.064)	**1.01×10^-5^**	0.046 (0.026 to 0.066)	**1.35×10^-5^**	0.042 (0.024 to 0.060)	**3.30 ×10^-6^**
wGPS-BCAMS(no CDH13)	0.046 (0.028 to 0.064)	**9.40×10^-7^**	0.045 (0.025 to 0.065)	**6.51×10^-6^**	0.04 (0.022 to 0.058)	**3.27×10^-6^**

a Adiponectin levels were natural logarithm transformed for analysis.

b Prepuberty: Tanner stage I; Midpuberty: Tanner stage II-III; Postpuberty: Tanner stage ≥ IV.

c Results are adjusted for age, sex, and BMI.

Boldface type indicates nominally significant values (P < 0.05).

### Impact of Lifestyle at Different Pubertal Stages

Controlling for age and sex, both exercise (P = 0.049) and walking to school (P = 4.47×10^-12^) was associated with increased adiponectin levels; adjustment for BMI attenuated the association of adiponectin levels with both exercise (P = 0.750) and walking to school (P = 1.07×10^-4^). Six diet items (breakfast, meat, dairy, soft drink, fried food, and snack) were significantly associated with adiponectin levels in the model adjusted for age and sex (soft drink, fried food, and snack), or in the model further adjusted for BMI (breakfast, meat, dairy, and soft drink) (**Table 3**). To better analyze the association of adiponectin with dietary structure, we used the six dietary items shown to be associated with adiponectin in either adjusted model to construct a new diet score. The diet score showed a stronger association with increased adiponectin levels than any single diet factor in both models with (*P* = 1.25 × 10^-8^) or without (*P* = 6.32 × 10^-6^) adjustment for BMI (**Supplementary Table S3**). Further analyses of lifestyle factors and gene-by-environment interactions will only focus on nine lifestyle factors that were associated with adiponectin levels in either adjustment model, including walking to school, exercise, diet score, and consumption of breakfast, meat, dairy, soft drink, fried food, and snacks. Further analyses (depicted in **Figure 2B** and summarized in **Supplementary Table S3**) adjusted for age and sex revealed different effects of the nine lifestyle factors on adiponectin levels among the three pubertal stages. However, none of the dietary items nor the diet score was associated with adiponectin levels at midpuberty. Walking to school was the only factor that influenced adiponectin levels throughout puberty [prepuberty: β = 0.114 (0.043, 0.185), *P =* 0.002; midpuberty: β = 0.077 (0.003, 0.151), *P* = 0.044; postpuberty: β = 0.203 (0.142, 0.264), *P* < 0.001]. In addition, exercise frequency was only associated with adiponectin levels after the onset of puberty [midpuberty: β = 0.031 (0.002, 0.060), *P* = 0.036; postpuberty: β = 0.025 (0.001, 0.049), *P* = 0.031]. After further adjustment for BMI, most of the effects of lifestyle factors on adiponectin levels were weakened, except for that of breakfast frequency, which decreased adiponectin levels significantly at postpuberty [β = -0.039 (-0.059 to -0.019), *P* = 0.003] (**Supplementary Table S3**). Additionally, none of the lifestyle factors, including walking to school, significantly affected adiponectin levels at midpuberty independent of BMI.

**Table 3:** Association of lifestyles factors with adiponectin levels.

Variables	Model 1- unadjusted		Model 2- adjusted for age and sex		Model 3- adjusted for age, sex and BMI.	
	β (95% CI)	P-value	β (95% CI)	P-value	β (95% CI)	P-value
Age	-0.049 (-0.055 to -0.043)	**3.12×10^-53^**	/	/	/	/
Sex	0.094 (0.055 to 0.133)	**2.69×10^-6^**	/	/	/	/
BMI (kg/m^2^)	-0.048 (-0.052 to 0.044)	**3.23×10^-132^**	-0.041 (-0.045 to -0.037)	**2.30×10^-87^**	/	/
Tanner stage	-0.097 (-0.111 to -0.083)	**5.82×10^-48^**	-0.076 (-0.100 to 0.052)	**6.90×10^-10^**	-0.025 (-0.049 to -0.001)	**0.040**
Exercise	0.026 (0.010 to 0.042)	**0.001**	0.015 (-0.001 to 0.031)	**0.049**	0.002 (-0.012 to 0.016)	0.75
Walking to school	0.183 (-0.209 to 0.575)	**2.33×10^-19^**	0.137 (0.098 to 0.176)	**4.47×10^-12^**	0.074 (0.037 to 0.111)	**1.07×10^-4^**
Sleep time	0.112 (0.077 to 0.147)	**2.17×10^-10^**	0.009 (-0.026 to 0.044)	0.641	-0.011 (-0.046 to 0.024)	0.531
**Diet factors**						
Breakfast	0.013 (-0.003 to 0.029)	0.095	-0.005 (-0.021 to 0.011)	0.479	-0.019 (-0.033 to -0.005)	**0.009**
Bean	0.012 (-0.002 to 0.026)	0.115	0.006 (-0.008 to 0.020)	0.375	-0.004 (-0.018 to 0.010)	0.532
Meat	-0.013 (-0.027 to 0.001)	0.063	-0.009 (-0.023 to 0.005)	0.204	-0.014 (-0.026 to -0.002)	**0.024**
Sea food	0.020 (0.004 to 0.036)	**0.014**	0.008 (-0.008 to 0.024)	0.328	-0.005 (-0.019 to 0.009)	0.537
Diary	0.006 (-0.006 to 0.018)	0.335	-0.010 (-0.022 to 0.002)	0.128	-0.019 (-0.031 to -0.007)	**0.002**
Vegetable	-0.012 (-0.039 to 0.015)	0.387	-0.007 (-0.032 to 0.018)	0.573	-0.003 (-0.027 to 0.021)	0.819
Fruit	0.003 (-0.013 to 0.019)	0.717	-0.004 (-0.018 to 0.010)	0.563	-0.010 (-0.024 to 0.004)	0.147
Fast food	0.016 (-0.009 to 0.041)	0.213	0.004 (-0.020 to 0.028)	0.744	-0.011 (-0.033 to 0.011)	0.352
Soft drink	-0.032 (-0.046 to -0.018)	**5.07×10^-6^**	-0.016 (-0.030 to -0.002)	**0.017**	-0.018 (-0.030 to -0.006)	**0.004**
Fried food	0.014 (-0.027 to 0.055)	0.523	0.018 (0.002 to 0.034)	**0.026**	0.014 (-0.001 to 0.029)	0.072
Snacks	0.049 (-0.343 to 0.441)	**0.017**	0.021 (0.008 to 0.034)	**0.001**	0.011 (-0.001 to 0.023)	0.072

Adiponectin levels were natural logarithm transformed (ln, e-based) for analysis.

Boldface type indicates nominally significant values (P < 0.05).

### SNPs-Lifestyle Interactions Across Pubertal Stages

**Figure 3:**
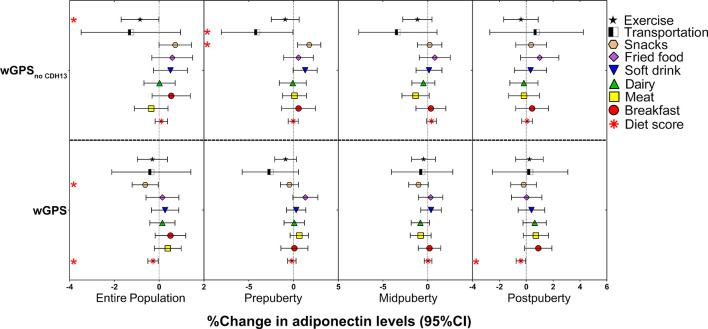
Effects of the interactions of the weighted genetic score with lifestyle factors on the % change in adiponectin levels according to the different puberty stages. The figure shows the effect and 95% CI of the interactions of wGPS_all_ and wGPS_no CDH13_ with lifestyle factors on adiponectin levels (% change in adiponectin levels per wGPS_all_ or wGPS_no CDH13_ per diet score or per lifestyle factors assigned score increase) in the entire population of children at different puberty stages. The results for the diet items were adjusted for age and sex. The results for the diet score were adjusted for age, sex, and activities (including exercise and transportation type). The results for the activities were adjusted for age, sex, and diet score; * *P* < 0.05.

After controlling for age and sex (**Supplementary Table S4**), we found two statistically significant interactions in the entire population: one between *WDR11-FGFR2*-rs3943077 and walking to school [β = -0.079 (-0.132 to -0.026), *P =* 0.004] and the other between *ADIPOQ*-rs6773957 and exercise [β = 0.038 (0.018 to 0.058), *P =* 1.71 × 10^-4^], as well as five nominally significant interactions. Regarding the interactions between GPS and lifestyle factors, as depicted in **Figure 3** and **Supplementary Table S5**, we identified nominally significant negative interactions of wGPS_all_ with diet score [β = -0.003 (-0.005 to 0.000), *P =* 0.022] and wGPS_no CDH13_ with exercise [β = -0.009 (-0.017 to 0.000), *P =* 0.049] and snacks [β = -0.006 (-0.012 to 0.000), *P =* 0.038] when age, sex and other lifestyle factors were controlled. The interaction between wGPS_all_ and diet score was more prominent at postpuberty than at other puberty stages. Further adjustment for BMI did not change the significance of the interactions between wGPS_no CDH13_ and exercise and snacks in the entire population and between wGPS_all_ and diet score in children at postpuberty (**Supplementary Tables S4, S5**).

**Figure 4:**
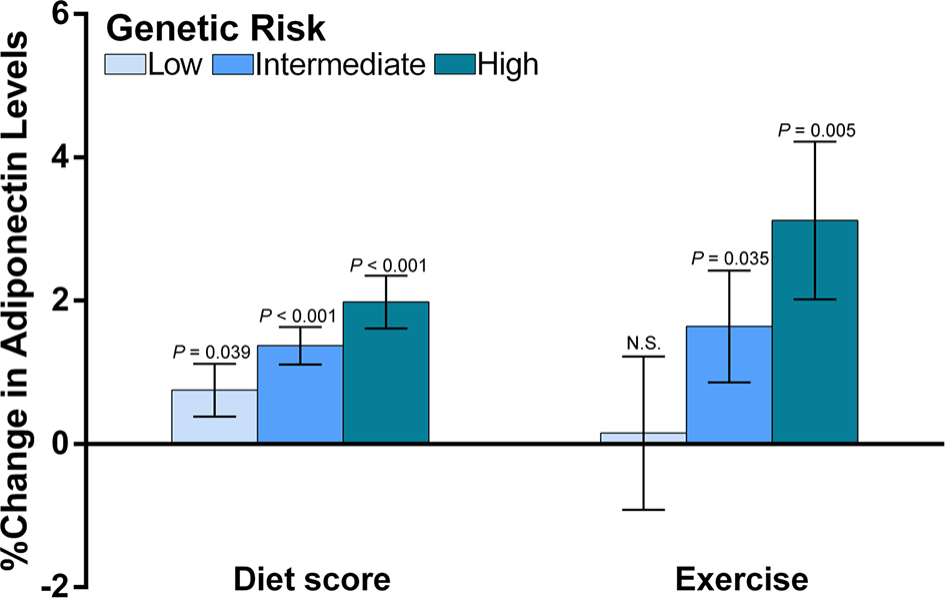
The associations of diet and exercise with adiponectin levels according to the categories of genetic risk. The figure shows the main effects (histograms) and SEs (error bars) of diet score and exercise on adiponectin levels (% change in adiponectin levels per assigned score increase for diet and exercise) according to the genetic risk for decreased adiponectin levels. The data for diet scores were adjusted for age, sex, transportation type and exercise. The data for exercise were adjusted for age, sex and diet score. As we reported an interaction between diet score and wGPSall and an interaction for exercise with wGPSno CDH13, we used wGPSall to identify the genetic modification of diet effect and used wGPSno CDH13 to identify the genetic modification of the exercise effect. Genetic risk was divided into low genetic risk (wGPSno CDH13 or wGPSall > mean +1SD), intermediate genetic risk (wGPSno CDH13 or wGPSall ≥ mean -1SD but ≤ mean+1SD) and high genetic risk (wGPSno CDH13 or wGPSall < mean-1SD). N.S. means the effect of exercise at low genetic risk is not significant.

As we identified an interaction between wGPS_all_ and diet score, and an interaction of wGPS_no CDH13_ with exercise, further stratified analyses were undertaken to observe effect modification. We firstly analyzed the associations of adiponectin levels with diet score and exercise according to the categories of genetic risk (**Figure 4**). We found that diet score and exercise had greater effects in children with a higher genetic risk for low adiponectin (**Figure 4**).

**Figure 5:**
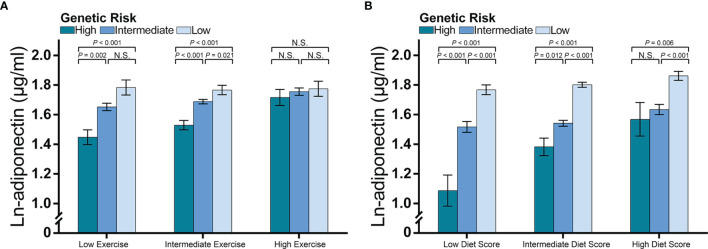
Adiponectin levels according to genetic risk and categories of diet and exercise. The figure shows multivariable-adjusted means (histograms) and SEs (error bar) of the natural logarithm transformed adiponectin levels according to the categories of lifestyle and genetic risk for decreased adiponectin levels. The P-values are the results of an ANCOVA comparing the adiponectin levels among the genetic risk groups. Data for exercise were adjusted for age, sex, and diet score, while data for diet were adjusted for age, sex, and exercise frequency. As we reported an interaction between diet score and wGPSall and an interaction for exercise with wGPSno CDH13, we used **(A)** wGPSall to identify genetic risk categories in the diet subgroups and **(B)** used wGPSno CDH13 to identify genetic risk categories in the exercise subgroups. Genetic risk was divided into low genetic risk (wGPSno CDH13 or wGPSall > mean + 1SD), intermediate genetic risk (wGPSno CDH13 or wGPSall ≥ mean - 1SD but ≤ mean + 1SD) and high genetic risk (wGPSno CDH13 or wGPSall < mean - 1SD). Similarly, diet and exercise were divided into high (diet score> mean + 1SD; exercise frequency ≤ 2 times per week), intermediate (diet score ≥ mean - 1SD but ≤ mean + 1SD; exercise frequency = 3-4 times per week), and low (diet score < mean – 1SD; exercise frequency ≥ 5 times/week). N.S. means the difference is not significant.

Second, we compared adiponectin concentrations between different categories of genetic risk and lifestyle levels (**Figure 5**). The difference in adiponectin levels between children at high genetic risk and those at intermediate or low genetic risk was more prominent among children with low and intermediate exercise levels than among those with high exercise levels (**Figure 5A**), whereas no significant difference in adiponectin levels was found among the genetic risk groups when the exercise frequency was high. A similar pattern was observed for diet levels, while an increased diet score significantly attenuated the difference in adiponectin levels between the high genetic risk group and intermediate genetic risk group (**Figure 5B**).

## Discussion

In this large cohort of Chinese children, we observed that eight lifestyle factors (breakfast, meat, dairy, soft drink, fried food, and snack consumption, walking to school, and exercise), and reported for the first time that five loci (*ADIPOQ*-rs10937273, *ADIPOQ*-rs6773957, *CDH13*-rs4783244, *WDR11-FGFR2*-rs3943077, and *PEPD*-rs889140) and two weighted polygene scores (wGPS_no CDH13_ and wGPS_all_), and some of their interactions were associated with adiponectin levels of children. We noted that the effects of a healthy diet and physical activity were more prominent in children at higher genetic risk of hypoadiponectinemia. We further found that the effects of these factors on adiponectin levels varied between pubertal stages, suggesting a modulating effect.

The value of identifying the associations between SNPs and adiponectin is related not only to the prediction of disease but also to the identification of causal steps on the path from genes to disease that can be targeted to reduce the risk (31). Previous GWASs have identified several genetic variants associated with adiponectin levels in adults (12, 13). Studies have shown that decreased androgen levels (32), decreased adipocyte size growth, better adipose tissue differentiation (5), and less visceral fat accumulation (33, 34), for which dramatic changes are observed during puberty, are associated with increased adiponectin levels (15, 35, 36). However, the relationships between these SNPs and adiponectin in childhood, and especially during puberty, a critical time for adipocyte hypertrophy and hyperplasia (37–39), are unclear. In line with GWASs conducted in adults, we found that *ADIPOQ*-rs10937273, *ADIPOQ*-rs6773957, *CDH13*-rs4783244, *WDR11-FGFR2*-rs3943077, and *PEPD*-rs889140 were associated with adiponectin levels among school children. According to previous studies, ADIPOQ-rs10937273 and ADIPOQ-rs6773957 were two unlinked SNP of adiponectin gene, while CDH13-rs4783244, PEPD-rs889140, WDR11-FGFR2-rs3943077 were associated with the synthesis of adiponectin receptor T-cadherin, adipocyte hypertrophy, and adipocyte differentiation respectively, which were further related to adiponectin levels. Therefore, our study suggested that the effects of SNPs on adiponectin levels reflected the activation of specific pathways. Different from studies in adults, *CMIP*-rs2925979 (12), an SNP related to lipolysis (40), was not associated with adiponectin levels in this study. One possible explanation is that the major processes of adipose development in children are differentiation and hypertrophy, and the effect of lipolysis might be weak during puberty.

**Figure 6:**
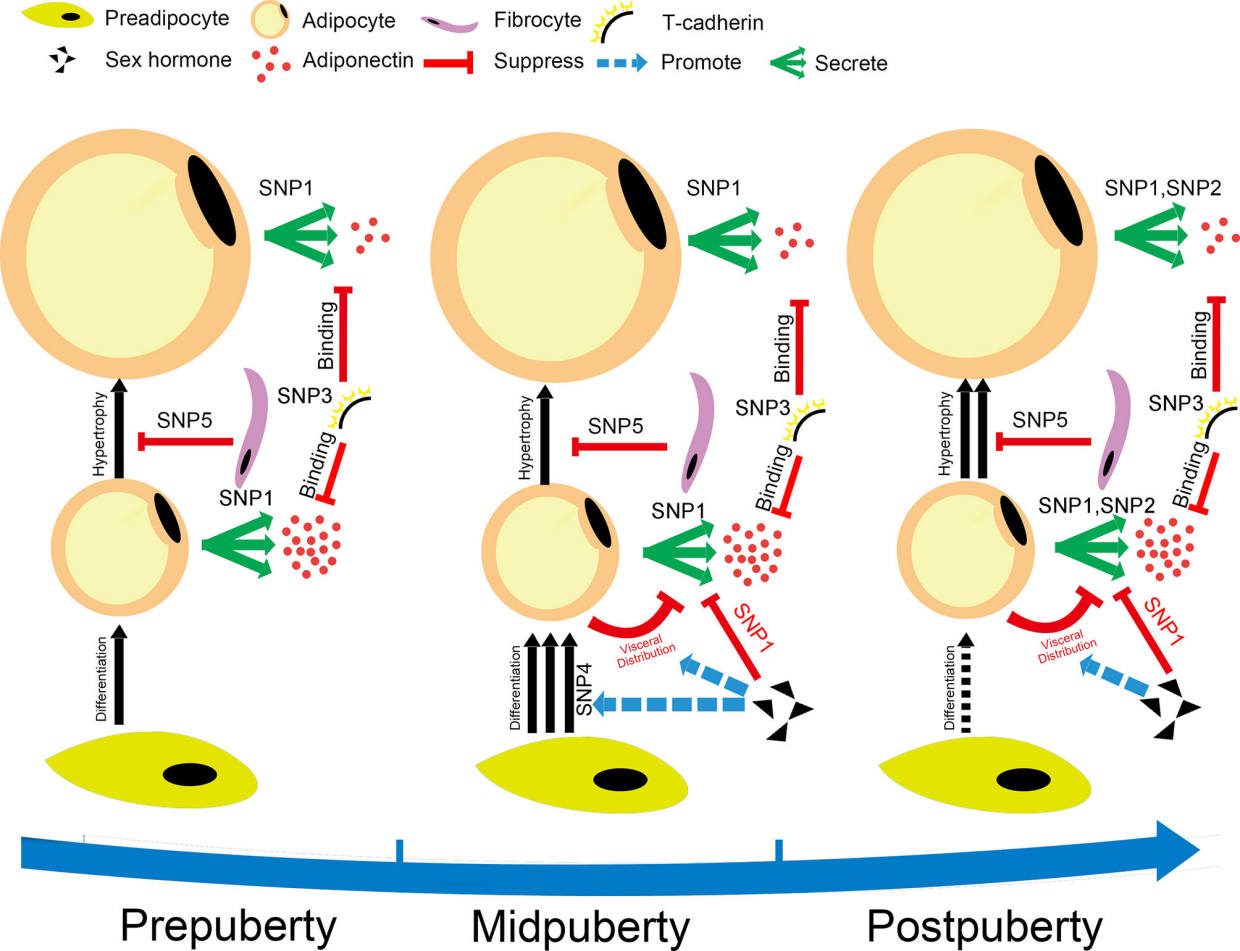
Hypothesis for the changes in adipocyte metabolism during puberty. The figure shows our hypothesis, which is that the modification effect of puberty on the SNP-adiponectin association is based on the development of adipose tissue during puberty. The SNPs shown in this figure are SNP1: *ADIPOQ*-rs10937273; SNP2: *ADIPOQ*-rs6773957; SNP3: *CDH13*-rs4783244; SNP4: *WDR11-FGFR2*-rs3943077; and SNP5: *PEPD*-rs889140. The puberty categories that we used in the current study were as follows: prepuberty (Tanner stage I), midpuberty (Tanner stage II-III), and postpuberty (Tanner stage ≥ IV). Prepuberty is a period during which the processes of puberty have not yet been activated completely. Midpuberty is the phase during which puberty has been activated but is not finished. Postpuberty is the stage at which the processes of puberty are nearly completed, and adolescents at postpuberty are similar to adults. The development of adipose tissue includes hyperplasia, related to *WDR11-FGFR2*-rs3943077, and the hypertrophy of adipocytes. The number of adipocytes increases quickly at midpuberty but remains relatively consistent after postpuberty. The size of adipocytes increases during puberty, which makes them secrete lower amounts of adiponectin. The process of hypertrophy is suppressed by the function of collagen, which might be related to *PEPD*-rs889140. *ADIPOQ*-rs10937273 and *ADIPOQ*-rs6773957 are SNPs located at different regulation sites of the adiponectin gene. They are activated at different puberty stages. Adiponectin is bound by T-cadherin encoded by *CDH13*, which is a high-molecular-weight adiponectin receptor expressed on target cells. Sex steroids decrease adiponectin levels by taking part in the regulation of both the distribution and differentiation of adipocytes.

To better understand the role of puberty in the SNP-adiponectin relationship, we divided puberty into three categories based on Tanner’s stages. We hypothesized that the modifying effect of puberty reflects the development of adipose tissue in children (**Figure 6**). We found that *PEPD*-rs889140, *ADIPOQ*-rs10937273, and *CDH13*-rs4783244 were significantly associated with adiponectin level both at prepuberty and in later life. Given that *PEPD*-rs889140, *ADIPOQ*-rs10937273, and *CDH13*-rs4783244 were related to collagen synthesis and adipocyte hypertrophy (41, 42), adiponectin, and adiponectin receptor T-cadherin (43, 44) respectively. Therefore, our findings suggested that adipocyte hypertrophy, the expression of both adiponectin and the adiponectin receptor are a continuous process throughout childhood. At midpuberty, the differences between sexes and the effect of *WDR11-FGFR2*-rs3943077, which is related to adipocyte differentiation (12), become significant, which supported that sex hormone levels are elevated and adipocyte differentiation is accelerated (37). At postpuberty, when the process of puberty is near its end, *ADIPOQ*-rs6773957, located in the 3’UTR of the adiponectin gene, is activated by unknown regulators.

Given that low adiponectin levels were associated with increased risk of metabolic disorders and cancers, findings of genetic and environmental factors related to adiponectin at a young age are important for early prevention and detection of these diseases. We found that both wGPS_all_ and wGPS_no CDH13_ were associated with increased adiponectin levels throughout puberty when adjusted for age, sex, and BMI. Therefore, children with a low wGPS_all_ or wGPS_no CDH13_ should pay more attention to their lifestyle to increase the adiponectin concentration in childhood.

In line with previous studies in both adults and children (14–17, 45–48), we found that lifestyle factors, including the consumption of breakfast, meat, dairy, soft drinks, fried food, and snacks and exercise, were associated with adiponectin levels. For the first time, we report that walking to school increases adiponectin levels in school-age children. Moreover, we identified several significant gene-by-environment interactions, suggesting that lifestyle factors affect adiponectin levels by activating a specific regulatory region of a gene containing an SNP. Growing evidence indicates that it is not only energy intake from food consumption but also special components of food that link the adiponectin-diet relationship (49, 50). The negative associations of adiponectin levels with dairy, meat, and breakfast consumption might be explained by the intake of vitamin D (49). Fried food may contribute to increased adiponectin levels by supplying fatty acids for adipocyte differentiation (50). The effect of snacking on increasing adiponectin levels might also be based on a greater intake of fatty acids. Sugar-sweetened beverage consumption has also been reported to cause an increased BMI and adipokine dysregulation, independent of energy intake (15, 16). Regarding activity factors, previous studies have shown discrepancies regarding the effects of exercise on adiponectin metabolism (48). Some studies indicate that exercise contributes to increased adiponectin levels only by causing weight loss, while others suggest that exercise itself can increase adiponectin levels independent of changes in body composition (48). Additionally, previous studies have indicated that different kinds of exercise might affect adiponectin levels through different mechanisms and that combining resistance exercise with aerobic exercise may be more beneficial (48). Similarly, we found that two activity factors, walking to school and regular exercise, were associated with increased adiponectin levels. However, the effect of exercise on adiponectin levels depended on BMI, while the influence of walking to school did not. Furthermore, we found that exercise and walking to school interacted with different SNPs significantly after adjusting for age, sex, and BMI: exercise interacted with *ADIPOQ*-rs6773957 while walking to school interacted with *WDR11-FGFR2*-rs3943077. Therefore, our results support the idea that different types of exercise affect adiponectin levels through various mechanisms (18).

In the current study, puberty also presented a strong modification effect on adiponectin-lifestyle associations. The modulation effect of puberty on adiponectin-lifestyle might also be explained by adipose tissue development during puberty. Whole-body growth is accelerated during puberty, and the effect of food consumption specifically on adipose tissue metabolism is relatively weak at midpuberty compared with the early and late puberty stage. However, activity factors are still effective methods for controlling weight at the midpuberty stage.

Our study had several strengths. The major advantages of our study include the large sample size of more than 3,400 participants and the completeness of the data, enabling us to analyze the influence of puberty on adiponectin modulation in a novel way. Previous studies addressing puberty and adiponectin have provided limited information regarding the possible mechanisms during this critical life period. The examination of the modifying impact of pubertal development on the effects of other factors led to the generation of several hypotheses regarding metabolic changes in adipocytes that warrant further investigation. Our study also had certain methodologic limitations. Firstly, because the current study was based on the BCAMS study, some detailed lifestyle information was not collected and the lifestyle indicators in this study were a little simple; for example, the components of the children’s breakfast were not recorded, which made it challenging to analyze some interactions between lifestyle factors. Besides, we only collected the consumption frequency of diet while not collected the amount of consumption of each diet item, and walking to school was recorded and simply reclassified into only two modes, ‘walking’ and ‘non-walking’. Secondly, although our 3,402-participant study cohort represents one of the largest pediatric cohorts examined to date, even a larger sample size is still required when analyzing data for different puberty subgroups. Thirdly, the ethnic background of human populations plays an important role in both genetic architecture and adiponectin levels; thus, our results cannot be directly generalized without further research in other ethnic groups. Lastly, the current study is a cross-sectional study, and it is therefore impossible to determine how the evaluated lifestyle factors will affect adiponectin levels in the future. Therefore, the examination of a larger prospective cohort with more comprehensive information is warranted to confirm the modulation effect of puberty stages.

## Conclusions

In this large pediatric cohort of a Chinese population, we found that associations of adiponectin with SNPs, lifestyle factors, and gene-by-environment interactions are modified by puberty stages. Children at high genetic risk might benefit more from dietary control, and exercise. The most important periods for diet control were shown to be the early and late stages of puberty, while exercise might be more important after the onset of puberty.

## Data Availability Statement

All datasets used in the current investigation are available from the corresponding author upon reasonable request.

## Ethics Statement

The studies involving human participants were reviewed and approved by the ethics committee of the Capital Institute of Pediatrics and is registered at www.clinicaltrails.gov (NCT03421444). The study followed the principles of the Declaration of Helsinki. Written informed consent to participate in this study was provided by the participants’ legal guardian/next of kin.

## Author Contributions

YW analyzed the data, drafted the manuscript. LZ and GL performed the data analyses and edited the manuscript. JF, YL, LL, LH, and QZ contributed to the BCAMS data collection. YG, XX, and LQ contributed to the data interpretation and reviewed the manuscript. SW contributed to the study design, data interpretation, and revised the manuscript. SG was responsible for the BCAMS follow-up study, contributed to the data interpretation and reviewed the manuscript. ML was responsible for the biomarker study in the BCAMS, contributed to the conception and design of the work and the acquisition and the interpretation of the data, and revised the manuscript. All authors contributed to the article and approved the submitted version.

## Funding

This work was supported by grants from the National Key Research Program of China (2016YFC1304801), the National Natural Science Foundation of China (81970732), the Capital Health Research and Development of Special (2020-2Z-40117), the Beijing Natural Science Foundation (7172169), the key program of Beijing Municipal Science & Technology Commission (D111100000611001, D111100000611002), the Beijing Science & Technology Star Program (2004A027), Novo Nordisk Union Diabetes Research Talent Fund (2011A002), the National Key Program of Clinical Science (WBYZ2011-873), the Non-profit Central Research Institute Fund of Chinese Academy of Medical Sciences (2017PT32020, 2018PT32001), the Key projects of medical school development of Shijingshan district (Beijing), and the CAMS Innovation Fund for Medical Sciences (CIFMS, 2021-1-I2M-016).

## Conflict of Interest

The authors declare that the research was conducted in the absence of any commercial or financial relationships that could be construed as a potential conflict of interest.

## Publisher’s Note

All claims expressed in this article are solely those of the authors and do not necessarily represent those of their affiliated organizations, or those of the publisher, the editors and the reviewers. Any product that may be evaluated in this article, or claim that may be made by its manufacturer, is not guaranteed or endorsed by the publisher.

## Abbreviations

BCAMS, Beijing Child and Adolescent Metabolic Syndrome; GWAS, Genome-wide association study; BMI, Body mass index; MS, Metabolic syndrome; TC, Total cholesterol; TG, Triglycerides; FBG, Fasting blood glucose; HDL, High-density lipoprotein; SNP, Single nucleotide polymorphism; GPS, Genetic predisposition scores; wGPS, Weighted genetic predisposition score; ANOVA, Analysis of variance; ANCOVA, Analysis of covariance; CI, Confidence interval.
