# Supplementary material for: Puberty Status Modifies the Effects of Genetic Variants, Lifestyle Factors and Their Interactions on Adiponectin: The BCAMS Study
Source: Front Endocrinol (Lausanne). 2021 Dec 24;12:737459. doi: 10.3389/fendo.2021.737459 (PMC8739496; doi:10.3389/fendo.2021.737459)

**Table S1.** **Comparison of lifestyle factors between subjects with risk of MS and without risk of MS**

| **Variables** | **With MS risk** | **Without MS risk** | **P-Value** |
| --- | --- | --- | --- |
| N (Male %) | 2112(52.7%) | 1290(46.1%) | ＜0.001 |
| Age (years) | 12±3 | 12±3 | 0.010 |
| BMI (kg/m2) | 23.9±4.7 | 18.8±3.4 | ＜0.001 |
| Normal weight, % | 597(27.6%) | 1047(78.8%) | ＜0.001 |
| Ln-adiponectin (μg/ml) ^a^ | 1.6±0.6 | 1.8±0.6 | ＜0.001 |
| Diet^b^ |  |  |  |
| Breakfast | 4.3±1.3 | 4.5±1.2 | 0.011 |
| Bean | 2.6±1.3 | 2.8±1.4 | ＜0.001 |
| Meat | 3.6±1.5 | 3.9±1.4 | ＜0.001 |
| Sea food | 2.1±1.2 | 2.3±1.3 | ＜0.001 |
| Diary | 3.7±1.6 | 3.9±1.5 | ＜0.001 |
| Vegetable | 4.8±0.7 | 4.8±0.8 | 0.745 |
| Fruit | 4.0±1.4 | 4.2±1.2 | ＜0.001 |
| Fast food | 1.4±0.7 | 1.5±0.8 | ＜0.001 |
| Soft drink | 2.4±1.5 | 2.5±1.5 | 0.225 |
| Fried food | 1.9±1.2 | 2.0±1.3 | 0.338 |
| Snacks | 2.5±1.5 | 2.7±1.6 | 0..055 |
| Exercise^b^ | 3.3±1.3 | 3.6±1.3 | ＜0.001 |
| Walking to school, % | 1071(50.0%) | 907(68.8%) | ＜0.001 |
| Sleep duration (h/day) | 8.5±1.1 | 8.6±1.2 | 0.064 |

a Adiponectin levels were natural logarithmically (ln) transformed.

b The values of the diet items and exercise were encoded as "seldom or never" = 1; "1 time/2 weeks" = 2; "1-2 times per week" = 3; "3-5 times per week" = 4; "> 5 times per week" = 5.

Data are expressed as the mean ± SD or n (%).

Abbreviation: MS, metabolic syndrome

With MS risk: based on at least one of the following criteria: (1) central obesity defined as waist circumference ≥ 90th percentile for age and gender; (2) elevated blood pressure defined as SBP/DBP ≥ 90th percentile for age, sex and height; (3) elevated TG defined as TG ≥ 1.24 mmol/L; (4) reduced HDL-C defined as ≤ 1.03 mmol/L; (5) hyperglycemia defined as FPG ≥ 5.6 mmol/L.

Without MS risk: not meet any of the above criteria.

**Table S2. Associations of the 6 SNPs and GPSs with adiponectin levels**

| **Gene** | **SNP** | **Position** | **Effect/non-effect alleles** | **EAF** | **P for HWE** | **β (95%CI)^a^** | ***P* ^a^** | **R^2^** |
| --- | --- | --- | --- | --- | --- | --- | --- | --- |
| *ADIPOQ* | rs10937273 | 5’ near gene | A^b^/G | A=0.41 | 0.27 | 0.074 (0.047 to 0.101) | **4.8×10^-8^** | 0.7% |
| *ADIPOQ* | rs6773957 | 3’ UTR | A^b^/ G | A=0.55 | 0.06 | 0.036 (0.011 to 0.061) | **0.007** | 0.2% |
| *CDH13* | rs4783244 | intron | T/G^b^ | T=0.33 | 0.53 | -0.202 (-0.229 to -0.175) | **8.28×10^-49^** | 5.2% |
| *WDR11-FGFR2* | rs3943077 | Between the 2 genes | A^b^/ G | A=0.60 | 0.36 | 0.044 (0.019 to 0.069) | **0.001** | 0.2% |
| *CMIP* | rs2925979 | intron | T/C^b^ | T=0.39 | 0.46 | -0.021 (-0.048 to 0.006) | 0.121 | <0.1% |
| *PEPD* | rs889140 | intron | A^b^/G | A=0.49 | 0.44 | 0.059 (0.034 to 0.084) | **8.19×10^-6^** | 0.5% |
| wGPS-GWAS^c^ | \ | \ | \ | \ | \ | 0.076 (0.068 to 0.085) | **1.09×10^-63^** | 6.8% |
| wGPS-BCAMS^c^ | \ | \ | \ | \ | \ | 0.073 (0.065 to 0.081) | **2.53×10^-65^** | 7.1% |
| wGPS-GWAS_no CDH13_^c^ | \ | \ | \ | \ | \ | 0.043 (0.031 to 0.055) | **1.59×10^-14^** | 1.8% |
| wGPS-BCAMS_no CDH13_^c^ | \ | \ | \ | \ | \ | 0.042 (0.032 to 0.052) | **1.83×10^-15^** | 1.9% |

Abbreviation: EAF, effect allele frequency; HWE, Hardy-Weinberg equilibrium;
^a^ The data were adjusted for age, sex, and BMI. Adiponectin levels were ln-transformed before analysis;
^b^ The alleles increased adiponectin levels;
^c^ Weighted genetic predisposition scores were created to account for differences in effect size by multiplying each SNP by its beta coefficient, which was derived from the published GWAS (wGPS-GWAS) from which we selected the six SNPs or from our data (wGPS-BCAMS). Two additional weighted risk scores were determined by excluding CDH13-rs4783244 (wGPS-GWAS_no CDH13_ and wGPS-BCAMS_no CDH13_). The weighted GPSs were divided by the sum of β and rescaled within the theoretical range of unweighted scores before the statistical analysis.

**Table S3. Association of lifestyle factors with adiponectin levels at different puberty statuses**

| **Lifestyle factors and linear model** | **Entire Population** | |  | **Prepuberty** | |  | **Midpuberty** | |  | **Postpuberty** | |
| --- | --- | --- | --- | --- | --- | --- | --- | --- | --- | --- | --- |
|  | β (95%CI) | *P* |  | β (95%CI) | *P* |  | β (95%CI) | *P* |  | β (95%CI) | *P* |
| **Breakfast** |  |  |  |  |  |  |  |  |  |  |  |
| Model 1^a^ | -0.005(-0.021 to 0.011) | 0.479 |  | -0.022(-0.057 to 0.013) | 0.208 |  | 0.021(-0.008 to 0.050) | 0.163 |  | -0.018(-0.040 to 0.004) | 0.087 |
| Model 2^b^ | -0.019(-0.033 to -0.005) | **0.009** |  | -0.026(-0.059 to 0.007) | 0.117 |  | 0.000(-0.027 to 0.027) | 0.983 |  | -0.039(-0.059 to -0.019) | **0.003** |
|  |  |  |  |  |  |  |  |  |  |  |  |
| **Meat** |  |  |  |  |  |  |  |  |  |  |  |
| Model 1^a^ | -0.009(-0.023 to 0.005) | 0.204 |  | -0.003(-0.027 to 0.021) | 0.770 |  | 0.004(-0.021 to 0.029) | 0.761 |  | -0.005(-0.025 to 0.015) | 0.645 |
| Model 2^b^ | -0.014(-0.026 to -0.002) | **0.024** |  | -0.006(-0.028 to 0.016) | 0.564 |  | -0.001(-0.025 to 0.023) | 0.923 |  | -0.015(-0.035 to 0.005) | 0.123 |
|  |  |  |  |  |  |  |  |  |  |  |  |
| **Dairy** |  |  |  |  |  |  |  |  |  |  |  |
| Model 1^a^ | -0.010(-0.022 to 0.002) | 0.128 |  | -0.026(-0.051 to -0.001) | **0.043** |  | 0.013(-0.009 to 0.035) | 0.254 |  | -0.019(-0.037 to -0.001) | **0.040** |
| Model 2^b^ | -0.018(-0.030 to -0.006) | **0.003** |  | -0.023(-0.031 to -0.015) | 0.061 |  | 0.006(-0.014 to 0.026) | 0.626 |  | -0.034(-0.052 to -0.016) | **1.42×10^-4^** |
|  |  |  |  |  |  |  |  |  |  |  |  |
| **Soft drink** |  |  |  |  |  |  |  |  |  |  |  |
| Model 1^a^ | -0.016(-0.030 to -0.002) | **0.017** |  | -0.020(-0.044 to 0.004) | 0.110 |  | 0.004(-0.021 to 0.029) | 0.748 |  | -0.021(-0.041 to -0.001) | **0.039** |
| Model 2^b^ | -0.018(-0.030 to -0.006) | **0.004** |  | -0.013(-0.037 to 0.011) | 0.261 |  | -0.004(-0.028 to 0.020) | 0.751 |  | -0.027(-0.047 to -0.007) | **0.007** |
|  |  |  |  |  |  |  |  |  |  |  |  |
| **Fried Food** |  |  |  |  |  |  |  |  |  |  |  |
| Model 1^a^ | 0.018(0.002 to 0.034) | **0.026** |  | 0.010(-0.020 to 0.041) | 0.497 |  | 0.013(-0.017 to 0.042) | 0.404 |  | 0.027(0.003 to 0.051) | 0.030 |
| Model 2^b^ | 0.014(-0.001 to 0.029) | 0.072 |  | 0.012(-0.017 to 0.041) | 0.410 |  | 0.011(-0.017 to 0.039) | 0.454 |  | 0.020(-0.003 to 0.043) | 0.092 |
|  |  |  |  |  |  |  |  |  |  |  |  |
| **Snacks** |  |  |  |  |  |  |  |  |  |  |  |
| Model 1^a^ | 0.021(0.009 to 0.034) | **0.001** |  | 0.005(-0.018 to 0.028) | 0.660 |  | 0.013(-0.010 to 0.037) | 0.268 |  | 0.037(0.017 to 0.056) | **2.89×10**^-4^ |
| Model 2^b^ | 0.011(-0.001 to 0.023) | 0.072 |  | 0.003(-0.019 to 0.025) | 0.795 |  | 0.000(-0.022 to 0.022) | 0.982 |  | 0.026(0.006 to 0.045) | **0.008** |
|  |  |  |  |  |  |  |  |  |  |  |  |
| **Diet Score** |  |  |  |  |  |  |  |  |  |  |  |
| Model 1^a^ | 0.012(0.007 to 0.017) | **6.32×10^-6^** |  | 0.014(0.004 to 0.024) | **0.008** |  | -0.002(-0.012 to 0.008) | 0.693 |  | 0.017(0.010 to 0.025) | **7.43×10^-06^** |
| Model 2^b^ | 0.014(0.009 to 0.019) | **1.25×10^-8^** |  | 0.013(0.003 to 0.023) | **0.011** |  | 0.001(-0.008 to 0.010) | 0.837 |  | 0.021(0.014 to 0.029) | **7.28×10^-09^** |
|  |  |  |  |  |  |  |  |  |  |  |  |
| **Effective exercise** |  |  |  |  |  |  |  |  |  |  |  |
| Model 1^a^ | 0.015(-0.001 to 0.031) | **0.049** |  | -0.007(-0.034 to 0.020) | 0.639 |  | 0.031(0.002 to 0.060) | **0.036** |  | 0.025(0.001 to 0.049) | **0.031** |
| Model 2^b^ | 0.002(-0.012 to 0.016) | 0.750 |  | -0.008(-0.033 to 0.017) | 0.540 |  | 0.009(-0.018 to 0.036) | 0.531 |  | 0.012(-0.010 to 0.034) | 0.272 |
|  |  |  |  |  |  |  |  |  |  |  |  |
| **Walking to school** | |  |  |  |  |  |  |  |  |  |  |
| Model 1^a^ | 0.137(0.098 to 0.176) | **4.47×10^-12^** |  | 0.114(0.043 to 0.185) | **0.002** |  | 0.077(0.003 to 0.151) | **0.044** |  | 0.203(0.142 to 0.264) | **1.15×10^-11^** |
| Model 2^b^ | 0.074(0.037 to 0.111) | **1.07×10^-4^** |  | 0.072(0.003 to 0.141) | **0.037** |  | 0.019(-0.052 to 0.090) | 0.598 |  | 0.134(0.075 to 0.193) | **9.15×10^-6^** |

Prepuberty: Tanner stage I; Midpuberty: Tanner stage II-III; Postpuberty: Tanner stage ≥ IV.

Adiponectin levels were natural logarithm transformed for analysis.

^a^ Data in model 1 were adjusted for age and sex.

^b^ Data in model 2 were adjusted for age, sex, and BMI.

**Table S4. Effect of gene-by-environment interaction on adiponectin levels after adjustment for age and sex at different puberty statuses**

| SNP/Lifestyle factors | Entire Population^b^ | |  | Prepuberty^b^ | |  | Midpuberty^b^ | |  | Postpuberty^b^ | |
| --- | --- | --- | --- | --- | --- | --- | --- | --- | --- | --- | --- |
|  | β(95%CI)^a^ | *P^a^* |  | β(95%CI)^a^ | *P^a^* |  | β(95%CI )^a^ | *P^a^* |  | β(95%CI )^a^ | *P^a^* |
| ***ADIPOQ-* rs10937273** |  |  |  |  |  |  |  |  |  |  |  |
| Breakfast | 0.004(-0.018 to 0.026) | 0.735 |  | -0.013(-0.062 to 0.036) | 0.597 |  | -0.014(-0.055 to 0.027) | 0.493 |  | 0.013(-0.016 to 0.042) | 0.363 |
| Meat | -0.005(-0.023 to 0.013) | 0.623 |  | 0.003(-0.028 to 0.034) | 0.874 |  | -0.027(-0.064 to 0.010) | 0.150 |  | 0.005(-0.022 to 0.032) | 0.717 |
| Dairy | 0.010(-0.061 to 0.081) | 0.771 |  | -0.104(-0.257 to 0.049) | 0.183 |  | -0.072(-0.203 to 0.059) | 0.283 |  | 0.069(-0.035 to 0.173) | 0.197 |
| Soft drink | 0.010(-0.008 to 0.028) | 0.304 |  | 0.031(-0.004 to 0.066) | 0.076 |  | -0.015(-0.050 to 0.020) | 0.398 |  | 0.016(-0.011 to 0.043) | 0.255 |
| Fried food | 0.014(-0.008 to 0.036) | 0.221 |  | 0.019(-0.023 to 0.061) | 0.375 |  | 0.029(-0.013 to 0.071) | 0.181 |  | 0.019(-0.015 to 0.053) | 0.276 |
| Snacks | 0.023(0.005 to 0.041) | **0.011** |  | 0.055(0.023 to 0.086) | **0.001** |  | 0.006(-0.028 to 0.041) | 0.711 |  | 0.013(-0.015 to 0.040) | 0.372 |
| Diet score | 0.003(-0.004 to 0.010) | 0.365 |  | 0.010(-0.004 to 0.025) | 0.159 |  | 0.016(0.002 to 0.030) | **0.022** |  | -0.003(-0.014 to 0.007) | 0.519 |
| Effective exercise | -0.016(-0.038 to 0.006) | 0.140 |  | -0.046(-0.085 to -0.007) | **0.018** |  | -0.034(-0.075 to 0.007) | 0.102 |  | 0.008(-0.023 to 0.039) | 0.611 |
| Walking to school | 0.018(-0.037 to 0.073) | 0.507 |  | -0.046(-0.146 to 0.054) | 0.363 |  | -0.006(-0.110 to 0.098) | 0.907 |  | 0.049(-0.035 to 0.133) | 0.258 |
|  |  |  |  |  |  |  |  |  |  |  |  |
| ***ADIPOQ-*rs6773957** |  |  |  |  |  |  |  |  |  |  |  |
| Breakfast | -0.020(-0.042 to 0.002) | 0.057 |  | 0.016(-0.031 to 0.063) | 0.506 |  | -0.028(-0.067 to 0.011) | 0.168 |  | -0.032(-0.061 to -0.003) | **0.035** |
| Meat | 0.003(-0.015 to 0.021) | 0.739 |  | -0.031(-0.062 to 0.000) | 0.054 |  | 0.014(-0.021 to 0.049) | 0.411 |  | 0.013(-0.014 to 0.040) | 0.335 |
| Dairy | 0.021(-0.046 to 0.088) | 0.528 |  | 0.016(-0.137 to 0.169) | 0.836 |  | 0.053(-0.070 to 0.176) | 0.403 |  | 0.005(-0.093 to 0.103) | 0.928 |
| Soft drink | -0.011(-0.029 to 0.007) | 0.226 |  | -0.028(-0.061 to 0.005) | 0.112 |  | 0.014(-0.019 to 0.047) | 0.414 |  | -0.013(-0.040 to 0.014) | 0.348 |
| Fried food | -0.025(-0.046 to -0.004) | **0.022** |  | -0.046(-0.087 to -0.006) | **0.026** |  | -0.018(-0.056 to 0.021) | 0.377 |  | -0.022(-0.056 to 0.011) | 0.194 |
| Snacks | -0.012(-0.029 to 0.005) | 0.179 |  | -0.020(-0.052 to 0.011) | 0.206 |  | -0.013(-0.045 ti 0.019) | 0.416 |  | -0.008(-0.035 to 0.018) | 0.542 |
| Diet score | 0.002(-0.005 to 0.008) | 0.640 |  | 0.000(-0.014 to 0.014) | 0.999 |  | 0.007(-0.006 to 0.020) | 0.312 |  | 0.000(-0.010 to 0.010) | 0.994 |
| Effective exercise | 0.038(0.018 to 0.058) | **1.71×10^-04^** |  | 0.045(0.008 to 0.082) | **0.021** |  | 0.042(0.005 to 0.079) | **0.029** |  | 0.023(-0.006 to 0.052) | 0.133 |
| Walking to school | 0.010(-0.043 to 0.063) | 0.721 |  | 0.029(-0.069 to 0.127) | 0.561 |  | 0.040(-0.062 to 0.142) | 0.436 |  | -0.017(-0.099 to 0.065) | 0.677 |
|  |  |  |  |  |  |  |  |  |  |  |  |
| ***CDH13-* -rs4783244** |  |  |  |  |  |  |  |  |  |  |  |
| Breakfast | -0.004(-0.026 to 0.018) | 0.733 |  | 0.022(-0.029 to 0.073) | 0.397 |  | 0.015(-0.024 to 0.054) | 0.467 |  | -0.028(-0.057 to 0.001) | 0.068 |
| Meat | -0.015(-0.033 to 0.003) | 0.106 |  | -0.007(-0.040 to 0.026) | 0.690 |  | 0.009(-0.026 to 0.044) | 0.620 |  | -0.031(-0.060 to -0.002) | **0.034** |
| Dairy | -0.010(-0.081 to 0.061) | 0.786 |  | 0.027(-0.122 to 0.176) | 0.728 |  | 0.090(-0.037 to 0.217) | 0.166 |  | -0.095(-0.203 to 0.013) | 0.087 |
| Soft drink | 0.002(-0.016 to 0.020) | 0.844 |  | 0.024(-0.009 to 0.057) | 0.157 |  | -0.019(-0.054 to 0.016) | 0.290 |  | -0.008(-0.037 to 0.021) | 0.586 |
| Fried food | -0.006(-0.028 to 0.017) | 0.627 |  | -0.033(-0.076 to 0.009) | 0.123 |  | -0.008(-0.048 to 0.032) | 0.703 |  | -0.004(-0.039 to 0.032) | 0.840 |
| Snacks | 0.024(0.006 to 0.042) | **0.008** |  | 0.036(0.005 to 0.067) | **0.025** |  | 0.025(-0.008 to 0.059) | 0.139 |  | 0.006(-0.023 ti 0.034) | 0.697 |
| Diet score | 0.006(-0.001 to 0.014) | 0.079 |  | -0.003(-0.018 to 0.012) | 0.698 |  | 0.000(-0.013 to 0.013) | 0.983 |  | 0.015(0.004 to 0.025) | **0.007** |
| Effective exercise | -0.005(-0.025 to 0.015) | 0.605 |  | 0.018(-0.019 to 0.055) | 0.353 |  | -0.002(-0.041 to 0.037) | 0.936 |  | -0.023(-0.054 to 0.008) | 0.145 |
| Walking to school | -0.013(-0.068 to 0.042) | 0.627 |  | 0.071(-0.029 to 0.171) | 0.165 |  | -0.036(-0.138 to 0.066) | 0.496 |  | -0.041(-0.129 to 0.047) | 0.353 |
|  |  |  |  |  |  |  |  |  |  |  |  |
| ***WDR11-FGFR2*-rs3943077** |  |  |  |  |  |  |  |  |  |  |  |
| Breakfast | 0.004(-0.018 to 0.026) | 0.725 |  | -0.020(-0.065 to 0.025) | 0.384 |  | 0.010(-0.029 to 0.049) | 0.620 |  | 0.007(-0.022 to 0.036) | 0.623 |
| Meat | -0.003(-0.021 to 0.015) | 0.735 |  | -0.001(-0.034 to 0.032) | 0.964 |  | -0.023(-0.058 to 0.012) | 0.187 |  | 0.009(-0.018 to 0.036) | 0.532 |
| Dairy | -0.005(-0.074 to 0.064) | 0.896 |  | -0.060(-0.215 to 0.095) | 0.450 |  | -0.010(-0.133 to 0.113) | 0.880 |  | 0.016(-0.088 to 0.120) | 0.759 |
| Soft drink | 0.000(-0.018 to 0.018) | 0.968 |  | 0.019(-0.014 to 0.052) | 0.268 |  | -0.004(-0.037 to 0.029) | 0.823 |  | -0.013(-0.042 to 0.016) | 0.364 |
| Fried food | -0.004(-0.026 to 0.018) | 0.699 |  | -0.003(-0.046 to 0.040) | 0.889 |  | -0.012(-0.052 to 0.027) | 0.541 |  | 0.001(-0.033 to 0.034) | 0.970 |
| Snacks | 0.007(-0.011 to 0.024) | 0.455 |  | 0.023(-0.009 to 0.056) | 0.159 |  | 0.014(-0.018 to 0.047) | 0.384 |  | -0.013(-0.041 to 0.015) | 0.361 |
| Diet score | 0.000(-0.007 to 0.007) | 0.939 |  | 0.004(-0.011 to 0.018) | 0.619 |  | 0.004(-0.009 to 0.018) | 0.532 |  | -0.003(-0.013 to 0.007) | 0.591 |
| Effective exercise | -0.013(-0.033 to 0.007) | 0.215 |  | -0.007(-0.044 to 0.030) | 0.706 |  | -0.029(-0.068 to 0.010) | 0.149 |  | -0.007(-0.038 to 0.024) | 0.686 |
| Walking to school | -0.079(-0.132 to -0.026) | **0.004** |  | -0.137(-0.235 to -0.039) | **0.006** |  | -0.087(-0.189 to 0.015) | 0.094 |  | -0.059(-0.143 to 0.025) | 0.177 |
|  |  |  |  |  |  |  |  |  |  |  |  |
| ***PEPD*-rs889140** |  |  |  |  |  |  |  |  |  |  |  |
| Breakfast | 0.009(-0.013 to 0.031) | 0.379 |  | -0.013(-0.062 to 0.036) | 0.597 |  | -0.014(-0.055 to 0.027) | 0.493 |  | 0.013(-0.016 to 0.042) | 0.363 |
| Meat | -0.009(-0.027 to 0.009) | 0.318 |  | 0.003(-0.028 to 0.034) | 0.874 |  | -0.027(-0.064 to 0.010) | 0.150 |  | 0.005(-0.022 to 0.032) | 0.717 |
| Dairy | 0.013(-0.056 to 0.082) | 0.717 |  | -0.104(-0.257 to 0.049) | 0.183 |  | -0.072(-0.203 to 0.059) | 0.283 |  | 0.069(-0.035 to 0.173) | 0.197 |
| Soft drink | 0.005(-0.013 to 0.023) | 0.617 |  | 0.031(-0.004 to 0.066) | 0.076 |  | -0.015(-0.050 to 0.020) | 0.398 |  | 0.016(-0.011 to 0.043) | 0.255 |
| Fried food | -0.008(-0.030 to 0.014) | 0.486 |  | -0.020(-0.061 to 0.020) | 0.322 |  | 0.004(-0.037 to 0.046) | 0.832 |  | -0.004(-0.039 to 0.031) | 0.833 |
| Snacks | -0.001(-0.019 to 0.017) | 0.925 |  | 0.008(-0.024 to 0.039) | 0.625 |  | -0.009(-0.043 to 0.024) | 0.580 |  | 0.002(-0.026 to 0.030) | 0.888 |
| Diet score | -0.002(-0.009 to 0.005) | 0.614 |  | -0.016(-0.030 to -0.002) | **0.023** |  | -0.007(-0.020 to 0.007) | 0.338 |  | 0.007(-0.003 to 0.018) | 0.164 |
| Effective exercise | 0.001(-0.021 to 0.023) | 0.909 |  | -0.046(-0.085 to -0.007) | **0.018** |  | -0.034(-0.075 to 0.007) | 0.102 |  | 0.008(-0.023 to 0.039) | 0.611 |
| Walking to school | -0.023(-0.076 to 0.030) | 0.405 |  | -0.046(-0.146 to 0.054) | 0.363 |  | -0.006(-0.110 to 0.098) | 0.907 |  | 0.049(-0.035 to 0.133) | 0.258 |
|  |  |  |  |  |  |  |  |  |  |  |  |
| **wGPS-BCAMS (no CDH13)** |  |  |  |  |  |  |  |  |  |  |  |
| Breakfast | 0.005(-0.003 to 0.014) | 0.196 |  | 0.005(-0.013 to 0.023) | 0.619 |  | 0.004(-0.012 to 0.020) | 0.611 |  | 0.006(-0.006 to 0.018) | 0.313 |
| Meat | -0.004(-0.011 to 0.003) | 0.287 |  | 0.003(-0.010 to 0.016) | 0.624 |  | -0.014(-0.028 to 0.001) | 0.065 |  | -0.002(-0.013 to 0.009) | 0.765 |
| Dairy | 0.000(-0.007 to 0.007) | 0.999 |  | -0.001(-0.015 to 0.014) | 0.938 |  | -0.006(-0.019 to 0.006) | 0.303 |  | 0.001(-0.009 to 0.011) | 0.878 |
| Soft drink | 0.005(-0.002 to 0.012) | 0.189 |  | 0.016(0.004 to 0.029) | **0.013** |  | -0.001(-0.015 to 0.013) | 0.876 |  | 0.002(-0.009 to 0.014) | 0.700 |
| Fried food | 0.005(-0.003 to 0.014) | 0.218 |  | 0.005(-0.011 to 0.020) | 0.574 |  | 0.009(-0.007 to 0.026) | 0.255 |  | 0.010(-0.004 to 0.023) | 0.160 |
| Snacks | 0.008(0.001 to 0.015) | **0.026** |  | 0.020(0.008 to 0.033) | **0.001** |  | 0.003(-0.010 to 0.016) | 0.656 |  | 0.003(-0.008 to 0.014) | 0.628 |
| Diet score | 0.001(-0.002 to 0.004) | 0.323 |  | 0.000(-0.006 to 0.005) | 0.933 |  | 0.004(-0.001 to 0.010) | 0.133 |  | 0.001(-0.003 to 0.005) | 0.590 |
| Effective exercise | -0.009(-0.017 to -0.001) | **0.022** |  | -0.011(-0.025 to 0.004) | 0.164 |  | -0.015(-0.030 to 0.001) | 0.066 |  | -0.006(-0.018 to 0.007) | 0.369 |
| Walking to school | -0.010(-0.031 to 0.011) | 0.354 |  | -0.037(-0.075 to 0.002) | 0.061 |  | -0.030(-0.071 to 0.012) | 0.158 |  | 0.010(-0.024 to 0.043) | 0.564 |
|  |  |  |  |  |  |  |  |  |  |  |  |
| **wGPS-BCAMS** |  |  |  |  |  |  |  |  |  |  |  |
| Breakfast | 0.003(-0.003 to 0.010) | 0.340 |  | 0.000(-0.015 to 0.014) | 0.953 |  | 0.000(-0.012 to 0.012) | 0.965 |  | 0.008(-0.001 to 0.018) | 0.095 |
| Meat | 0.004(-0.002 to 0.009) | 0.214 |  | 0.007(-0.003 to 0.016) | 0.178 |  | -0.007(-0.018 to 0.004) | 0.189 |  | 0.008(-0.001 to 0.017) | 0.092 |
| Dairy | 0.000(-0.005 to 0.006) | 0.874 |  | -0.002(-0.013 to 0.009) | 0.692 |  | -0.007(-0.017 to 0.003) | 0.155 |  | 0.006(-0.002 to 0.015) | 0.122 |
| Soft drink | 0.002(-0.004 to 0.007) | 0.582 |  | 0.001(-0.009 to 0.011) | 0.861 |  | 0.002(-0.008 to 0.013) | 0.651 |  | 0.005(-0.004 to 0.015) | 0.274 |
| Fried food | 0.004(-0.003 to 0.011) | 0.233 |  | 0.013(0.000 to 0.026) | 0.053 |  | 0.008(-0.005 to 0.021) | 0.225 |  | 0.004(-0.007 to 0.015) | 0.506 |
| Snacks | -0.004(-0.010 to 0.001) | 0.131 |  | -0.003(-0.012 to 0.007) | 0.582 |  | -0.007(-0.018 to 0.003) | 0.168 |  | -0.001(-0.010 to 0.008) | 0.850 |
| Diet score | -0.002(-0.004 to 0.001) | 0.181 |  | -0.001(-0.005 to 0.004) | 0.790 |  | 0.001(-0.003 to 0.005) | 0.501 |  | -0.004(-0.007 to 0.000) | **0.030** |
| Effective exercise | -0.004(-0.010 to 0.003) | 0.242 |  | -0.011(-0.022 to 0.001) | 0.071 |  | -0.006(-0.018 to 0.007) | 0.374 |  | 0.002(-0.008 to 0.012) | 0.665 |
| Walking to school | -0.001(-0.018 to 0.016) | 0.914 |  | -0.027(-0.057 to 0.003) | 0.079 |  | -0.003(-0.035 to 0.029) | 0.860 |  | 0.011(-0.016 to 0.038) | 0.425 |

^a^ The coefficient(β) and P value of linear regression evaluating the effect of gene-by-environment interactions on Ln-adiponectin (adiponectin levels were natural logarithm transformed) adjusted for age and sex.

^b^ Prepuberty: Tanner stage I; Midpuberty: Tanner stage II-III; Postpuberty: Tanner stage ≥ IV.

| **Table S5. Effect of gene-by-environment interaction on adiponectin levels after adjustment for age, sex, and BMI at different puberty statuses** | | | | | | | | | | | |
| --- | --- | --- | --- | --- | --- | --- | --- | --- | --- | --- | --- |
| SNP/Lifestyle factors | Entire Population^b^ | |  | Prepuberty^b^ | |  | Midpuberty^b^ | |  | Postpuberty^b^ | |
|  | β(95%CI)^a^ | P^a^ |  | β(95%CI)^a^ | P^a^ |  | β(95%CI )^a^ | P^a^ |  | β(95%CI )^a^ | P^a^ |
| ***ADIPOQ-* rs10937273** |  |  |  |  |  |  |  |  |  |  |  |
| Breakfast | 0.002(-0.020 to 0.023) | 0.870 |  | -0.014(-0.065 to 0.038) | 0.606 |  | -0.020(-0.063 to 0.022) | 0.354 |  | 0.010(-0.020 to 0.040) | 0.513 |
| Meat | -0.004(-0.023 to 0.015) | 0.693 |  | -0.003(-0.037 to 0.030) | 0.841 |  | -0.030(-0.069 to 0.008) | 0.125 |  | 0.008(-0.021 to 0.037) | 0.574 |
| Dairy | 0.017(-0.056 to 0.091) | 0.649 |  | -0.112(-0.272 to 0.049) | 0.173 |  | -0.017(-0.155 to 0.121) | 0.812 |  | 0.036(-0.074 to 0.145) | 0.526 |
| Soft drink | 0.009(-0.010 to 0.029) | 0.342 |  | 0.024(-0.013 to 0.060) | 0.202 |  | -0.006(-0.043 to 0.031) | 0.747 |  | 0.015(-0.014 to 0.044) | 0.315 |
| Fried food | 0.013(-0.010 to 0.037) | 0.273 |  | 0.017(-0.027 to 0.062) | 0.441 |  | 0.018(-0.027 to 0.063) | 0.440 |  | 0.024(-0.012 to 0.059) | 0.194 |
| Snacks | 0.020(0.002 to 0.039) | 0.032 |  | 0.049(0.016 to 0.082) | **0.004** |  | 0.005(-0.031 to 0.041) | 0.803 |  | 0.010(-0.018 to 0.039) | 0.478 |
| Diet score | 0.002(-0.005 to 0.010) | 0.512 |  | 0.011(-0.004 to 0.026) | 0.138 |  | 0.012(-0.002 to 0.027) | 0.098 |  | -0.002(-0.013 to 0.009) | 0.705 |
| Effective exercise | -0.012(-0.034 to 0.009) | 0.258 |  | -0.042(-0.082 to -0.002) | **0.040** |  | -0.029(-0.072 to 0.014) | 0.184 |  | 0.009(-0.024 to 0.041) | 0.593 |
| Walking to school | 0.009(-0.048 to 0.066) | 0.752 |  | -0.059(-0.163 to 0.045) | 0.267 |  | -0.017(-0.128 to 0.093) | 0.756 |  | 0.039(-0.049 to 0.128) | 0.382 |
|  |  |  |  |  |  |  |  |  |  |  |  |
| ***ADIPOQ-*rs6773957** |  |  |  |  |  |  |  |  |  |  |  |
| Breakfast | -0.021(-0.043 to 0.001) | 0.061 |  | 0.011(-0.038 to 0.060) | 0.651 |  | -0.028(-0.070 to 0.014) | 0.192 |  | -0.031(-0.062 to 0.000) | 0.053 |
| Meat | 0.000(-0.018 to 0.019) | 0.976 |  | -0.028(-0.061 to 0.005) | 0.099 |  | 0.012(-0.024 to 0.048) | 0.521 |  | 0.008(-0.021 to 0.036) | 0.600 |
| Dairy | 0.023(-0.047 to 0.093) | 0.523 |  | 0.042(-0.119 to 0.204) | 0.606 |  | 0.060(-0.071 to 0.191) | 0.370 |  | 0.024(-0.080 to 0.127) | 0.655 |
| Soft drink | -0.012(-0.031 to 0.007) | 0.201 |  | -0.014(-0.050 to 0.021) | 0.431 |  | 0.011(-0.024 to 0.047) | 0.536 |  | -0.020(-0.049 to 0.009) | 0.187 |
| Fried food | -0.028(-0.050 to -0.005) | **0.016** |  | -0.049(-0.092 to -0.006) | **0.025** |  | -0.015(-0.056 to 0.026) | 0.479 |  | -0.025(-0.060 to 0.010) | 0.162 |
| Snacks | -0.012(-0.030 to 0.006) | 0.195 |  | -0.014(-0.047 to 0.019) | 0.395 |  | -0.016(-0.050 to 0.018) | 0.371 |  | -0.011(-0.039 to 0.017) | 0.455 |
| Diet score | 0.001(-0.006 to 0.009) | 0.707 |  | 0.002(-0.013 to 0.017) | 0.757 |  | 0.007(-0.007 to 0.020) | 0.340 |  | 0.000(-0.011 to 0.010) | 0.941 |
| Effective exercise | 0.039(0.018 to 0.059) | **2.62×10^-4^** |  | 0.050(0.011 to 0.090) | **0.013** |  | 0.040(0.001 to 0.080) | **0.046** |  | 0.023(-0.008 to 0.054) | 0.153 |
| Walking to school | 0.020(-0.035 to 0.075) | 0.483 |  | 0.026(-0.076 to 0.129) | 0.619 |  | 0.047(-0.061 to 0.154) | 0.395 |  | -0.006(-0.090 to 0.079) | 0.896 |
|  |  |  |  |  |  |  |  |  |  |  |  |
| ***CDH13-* -rs4783244** |  |  |  |  |  |  |  |  |  |  |  |
| Breakfast | -0.010(-0.032 to 0.012) | 0.373 |  | 0.018(-0.036 to 0.071) | 0.517 |  | 0.008(-0.035 to 0.051) | 0.711 |  | -0.034(-0.066 to -0.002) | **0.035** |
| Meat | -0.015(-0.035 to 0.004) | 0.126 |  | -0.008(-0.043 to 0.026) | 0.628 |  | 0.013(-0.025 to 0.050) | 0.519 |  | -0.029(-0.060 to 0.001) | 0.059 |
| Dairy | -0.006(-0.024 to 0.012) | 0.506 |  | -0.004(-0.042 to 0.033) | 0.818 |  | 0.025(-0.007 to 0.058) | 0.128 |  | -0.029(-0.057 to -0.001) | **0.040** |
| Soft drink | -0.002(-0.021 to 0.018) | 0.861 |  | 0.010(-0.025 to 0.046) | 0.568 |  | -0.020(-0.057 to 0.017) | 0.287 |  | -0.002(-0.033 to 0.029) | 0.895 |
| Fried food | 0.005(-0.019 to 0.028) | 0.701 |  | -0.033(-0.078 to 0.012) | 0.149 |  | 0.006(-0.037 to 0.048) | 0.796 |  | 0.010(-0.028 to 0.047) | 0.609 |
| Snacks | 0.030(0.012 to 0.049) | **0.002** |  | 0.038(0.005 to 0.071) | **0.023** |  | 0.035(-0.001 to 0.070) | 0.056 |  | 0.011(-0.019 to 0.041) | 0.467 |
| Diet score | 0.010(0.003 to 0.018) | **0.008** |  | 0.003(-0.013 to 0.018) | 0.743 |  | 0.002(-0.012 to 0.016) | 0.791 |  | 0.017(0.006 to 0.028) | **0.003** |
| Effective exercise | -0.005(-0.027 to 0.016) | 0.626 |  | 0.012(-0.027 to 0.052) | 0.539 |  | 0.001(-0.041 to 0.042) | 0.974 |  | -0.022(-0.055 to 0.011) | 0.189 |
| Walking to school | -0.002(-0.021 to 0.018) | 0.861 |  | 0.010(-0.025 to 0.046) | 0.568 |  | -0.020(-0.057 to 0.017) | 0.287 |  | -0.002(-0.033 to 0.029) | 0.895 |
|  |  |  |  |  |  |  |  |  |  |  |  |
| ***WDR11-FGFR2*-rs3943077** |  |  |  |  |  |  |  |  |  |  |  |
| Breakfast | 0.000(-0.022 to 0.022) | 0.993 |  | -0.027(-0.074 to 0.021) | 0.277 |  | 0.016(-0.026 to 0.059) | 0.447 |  | 0.002(-0.028 to 0.033) | 0.891 |
| Meat | -0.004(-0.023 to 0.015) | 0.689 |  | 0.005(-0.030 to 0.040) | 0.778 |  | -0.021(-0.057 to 0.016) | 0.266 |  | 0.000(-0.029 to 0.029) | 0.998 |
| Dairy | -0.023(-0.096 to 0.050) | 0.534 |  | -0.072(-0.235 to 0.091) | 0.389 |  | -0.037(-0.168 to 0.094) | 0.578 |  | 0.002(-0.025 to 0.029) | 0.898 |
| Soft drink | -0.004(-0.023 to 0.015) | 0.682 |  | 0.022(-0.013 to 0.058) | 0.215 |  | -0.014(-0.050 to 0.022) | 0.449 |  | -0.015(-0.045 to 0.015) | 0.329 |
| Fried food | -0.009(-0.032 to 0.014) | 0.451 |  | 0.009(-0.036 to 0.054) | 0.700 |  | -0.019(-0.061 to 0.023) | 0.370 |  | -0.010(-0.045 to 0.025) | 0.567 |
| Snacks | 0.006(-0.013 to 0.024) | 0.533 |  | 0.025(-0.009 to 0.059) | 0.148 |  | 0.007(-0.027 to 0.041) | 0.697 |  | -0.010(-0.039 to 0.019) | 0.487 |
| Diet score | 0.002(-0.006 to 0.009) | 0.659 |  | 0.005(-0.011 to 0.020) | 0.544 |  | 0.005(-0.010 to 0.019) | 0.541 |  | -0.001(-0.012 to 0.010) | 0.857 |
| Effective exercise | -0.018(-0.040 to 0.003) | 0.094 |  | -0.011(-0.051 to 0.029) | 0.591 |  | -0.036(-0.078 to 0.005) | 0.088 |  | -0.010(-0.044 to 0.023) | 0.535 |
| Walking to school | -0.073(-0.129 to -0.017) | **0.011** |  | -0.142(-0.244 to -0.039) | **0.007** |  | -0.092(-0.199 to 0.016) | 0.094 |  | -0.047(-0.136 to 0.042) | 0.299 |
|  |  |  |  |  |  |  |  |  |  |  |  |
| ***PEPD*-rs889140** |  |  |  |  |  |  |  |  |  |  |  |
| Breakfast | 0.014(-0.008 to 0.036) | 0.210 |  | 0.050(0.002 to 0.099) | **0.043** |  | 0.003(-0.039 to 0.044) | 0.888 |  | -0.001(-0.032 to 0.031) | 0.967 |
| Meat | -0.010(-0.028 to 0.009) | 0.324 |  | -0.010(-0.044 to 0.024) | 0.574 |  | -0.010(-0.047 to 0.027) | 0.589 |  | -0.010(-0.040 to 0.019) | 0.488 |
| Dairy | 0.025(-0.047 to 0.096) | 0.503 |  | 0.132(-0.027 to 0.290) | 0.103 |  | 0.014(-0.116 to 0.144) | 0.829 |  | -0.014(-0.040 to 0.013) | 0.312 |
| Soft drink | 0.007(-0.012 to 0.027) | 0.461 |  | 0.029(-0.006 to 0.064) | 0.105 |  | 0.027(-0.009 to 0.064) | 0.139 |  | -0.013(-0.044 to 0.017) | 0.382 |
| Fried food | -0.003(-0.026 to 0.020) | 0.805 |  | -0.021(-0.063 to 0.021) | 0.326 |  | 0.017(-0.026 to 0.061) | 0.440 |  | -0.003(-0.040 to 0.034) | 0.876 |
| Snacks | -0.010(-0.045 to 0.026） | 0.595 |  | 0.004(-0.029 to 0.037） | 0.827 |  | -0.010(-0.045 to 0.026） | 0.595 |  | 0.008(-0.021 to 0.038） | 0.586 |
| Diet score | -0.003(-0.010 to 0.005) | 0.477 |  | -0.018(-0.033 to -0.003) | **0.016** |  | -0.006(-0.020 to 0.009) | 0.439 |  | 0.007(-0.004 to 0.018) | 0.184 |
| Effective exercise | 0.013(-0.009 to 0.034) | 0.255 |  | 0.044(0.003 to 0.085) | **0.038** |  | 0.000(-0.042 to 0.041) | 0.985 |  | -0.007(-0.040 to 0.025) | 0.655 |
| Walking to school | -0.023(-0.079 to 0.033) | 0.420 |  | -0.066(-0.168 to 0.036) | 0.202 |  | -0.034(-0.143 to 0.074) | 0.535 |  | 0.013(-0.074 to 0.101) | 0.763 |
|  |  |  |  |  |  |  |  |  |  |  |  |
| **wGPS-BCAMS(no CDH13)** |  |  |  |  |  |  |  |  |  |  |  |
| Breakfast | 0.005(-0.003 to 0.014) | 0.215 |  | 0.006(-0.013 to 0.024) | 0.562 |  | 0.004(-0.013 to 0.020) | 0.677 |  | 0.004(-0.008 to 0.017) | 0.484 |
| Meat | -0.004(-0.011 to 0.004) | 0.355 |  | 0.001(-0.012 to 0.014) | 0.876 |  | -0.014(-0.029 to 0.002) | 0.081 |  | -0.002(-0.013 to 0.010) | 0.772 |
| Dairy | 0.000(-0.007 to 0.007) | 0.955 |  | -0.001(-0.016 to 0.014) | 0.911 |  | -0.005(-0.018 to 0.008) | 0.462 |  | -0.002(-0.012 to 0.009) | 0.741 |
| Soft drink | 0.003(-0.003 to 0.009) | 0.395 |  | 0.003(-0.008 to 0.014) | 0.599 |  | 0.004(-0.008 to 0.015) | 0.524 |  | 0.004(-0.006 to 0.014) | 0.444 |
| Fried food | 0.006(-0.003 to 0.015) | 0.207 |  | 0.006(-0.011 to 0.022) | 0.507 |  | 0.008(-0.009 to 0.025) | 0.368 |  | 0.010(-0.004 to 0.024) | 0.168 |
| Snacks | 0.007(0.000 to 0.014) | 0.051 |  | 0.018(0.005 to 0.030) | **0.008** |  | 0.002(-0.011 to 0.016) | 0.753 |  | 0.004(-0.008 to 0.015) | 0.539 |
| Diet score | 0.001(-0.002 to 0.004) | 0.490 |  | 0.000(-0.006 to 0.005) | 0.894 |  | 0.004(-0.001 to 0.010) | 0.124 |  | 0.001(-0.003 to 0.005) | 0.755 |
| Effective exercise | -0.009(-0.017 to 0.000) | **0.049** |  | -0.009(-0.025 to 0.006) | 0.242 |  | -0.012(-0.028 to 0.005) | 0.165 |  | -0.004(-0.017 to 0.009) | 0.547 |
| Walking to school | -0.013(-0.035 to 0.010) | 0.266 |  | -0.041(-0.081 to -0.001) | 0.047 |  | -0.033(-0.077 to 0.010) | 0.135 |  | 0.008(-0.027 to 0.043) | 0.666 |
|  |  |  |  |  |  |  |  |  |  |  |  |
| **wGPS-BCAMS** |  |  |  |  |  |  |  |  |  |  |  |
| Breakfast | 0.005(-0.002 to 0.012) | 0.146 |  | 0.001(-0.014 to 0.016) | 0.899 |  | 0.002(-0.011 to 0.015) | 0.751 |  | 0.009(-0.001 to 0.019) | 0.084 |
| Meat | 0.004(-0.002 to 0.010) | 0.200 |  | 0.006(-0.004 to 0.017) | 0.219 |  | -0.008(-0.020 to 0.004) | 0.184 |  | 0.007(-0.002 to 0.017) | 0.137 |
| Dairy | 0.001(-0.004 to 0.007) | 0.607 |  | 0.001(-0.011 to 0.012) | 0.893 |  | -0.008(-0.018 to 0.002) | 0.116 |  | 0.006(-0.002 to 0.015) | 0.158 |
| Soft drink | 0.003(-0.003 to 0.009) | 0.395 |  | 0.003(-0.008 to 0.014) | 0.599 |  | 0.004(-0.008 to 0.015) | 0.524 |  | 0.004(-0.006 to 0.014) | 0.444 |
| Fried food | 0.001(-0.006 to 0.009) | 0.692 |  | 0.013(-0.001 to 0.027) | 0.065 |  | 0.003(-0.010 to 0.017) | 0.636 |  | 0.000(-0.011 to 0.012) | 0.959 |
| Snacks | -0.006(-0.012 to 0.000) | **0.038** |  | -0.005(-0.015 to 0.005) | 0.370 |  | -0.010(-0.021 to 0.001) | 0.065 |  | -0.002(-0.012 to 0.008) | 0.688 |
| Diet score | -0.003(-0.005 to 0.000) | **0.022** |  | -0.002(-0.006 to 0.003) | 0.426 |  | 0.000(-0.004 to 0.005) | 0.858 |  | -0.004(-0.008 to 0.000) | **0.026** |
| Effective exercise | -0.003(-0.010 to 0.004) | 0.380 |  | -0.009(-0.021 to 0.003) | 0.149 |  | -0.005(-0.018 to 0.009) | 0.494 |  | 0.002(-0.008 to 0.013) | 0.658 |
| Walking to school | -0.003(-0.021 to 0.014) | 0.699 |  | -0.026(-0.057 to 0.006) | 0.108 |  | -0.006(-0.040 to 0.028) | 0.723 |  | 0.003(-0.025 to 0.031) | 0.840 |

^a^ The coefficient(β) and P value of linear regression evaluating the effect of gene-by-environment interactions on Ln-adiponectin (adiponectin levels were natural logarithm transformed) adjusted for age, sex, and BMI.

^b^ Prepuberty: Tanner stage I; Midpuberty: Tanner stage II-III; Postpuberty: Tanner stage ≥ IV.


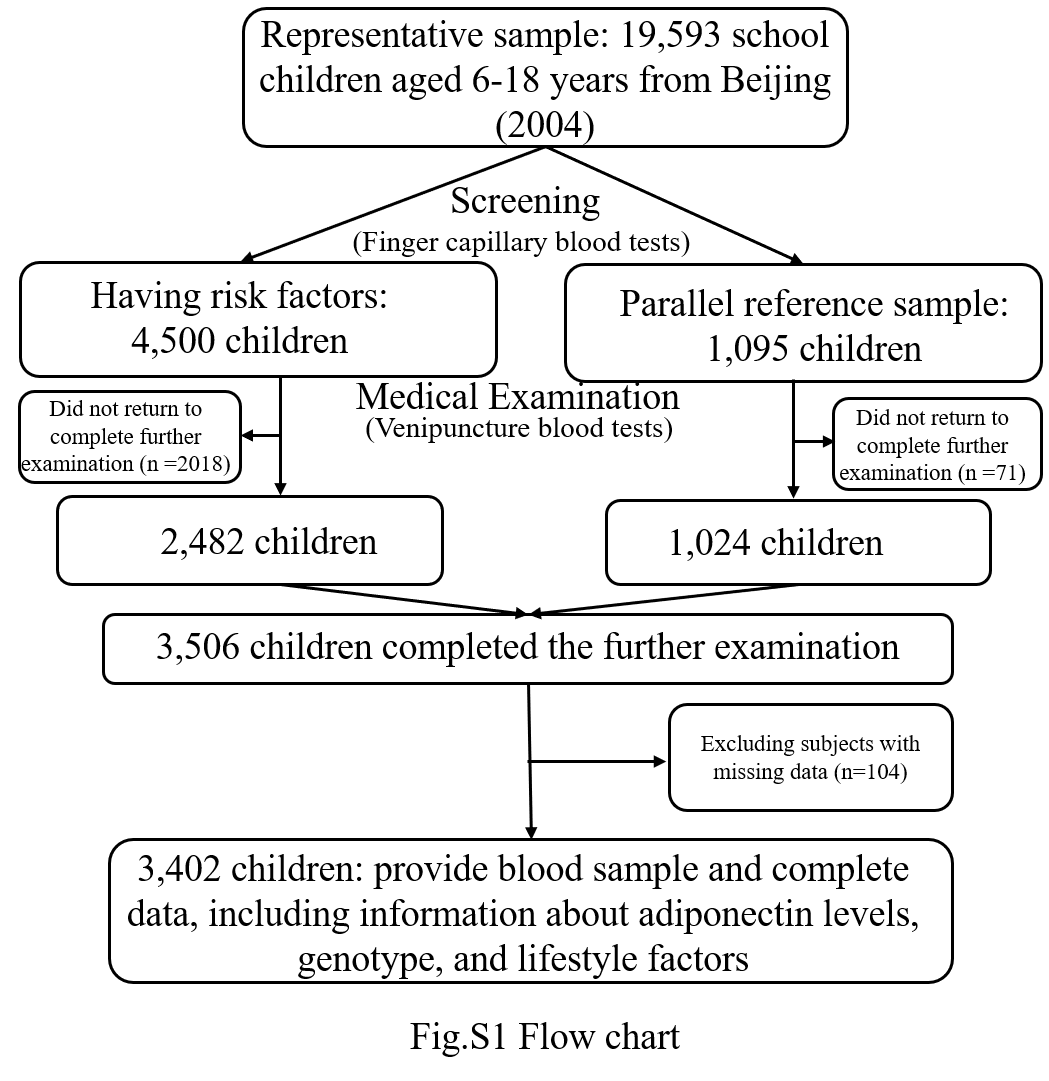

Supplement: Supplementary file 1 [file DataSheet_1.docx]
